# Comprehensive Assessment of Functional Effects of Commonly Used Sugar Substitute Sweeteners on *Ex Vivo* Human Gut Microbiome

**DOI:** 10.1128/spectrum.00412-22

**Published:** 2022-06-13

**Authors:** Zhongzhi Sun, Wenju Wang, Leyuan Li, Xu Zhang, Zhibin Ning, Janice Mayne, Krystal Walker, Alain Stintzi, Daniel Figeys

**Affiliations:** a School of Pharmaceutical Sciences, Faculty of Medicine, University of Ottawa, Ottawa, Ontario, Canada; b Shanghai Institute of Materia Medica-University of Ottawa Joint Research Center in Systems and Personalized Pharmacology, University of Ottawa, Ottawa, Ontario, Canada; c Ottawa Institute of Systems Biology, University of Ottawa, Ottawa, Ontario, Canada; d Department of Biochemistry, Microbiology, and Immunology, Faculty of Medicine, University of Ottawa, Ottawa, Ontario, Canada; University of Nevada Reno

**Keywords:** sugar substitute sweetener, gut microbiome, metaproteomics

## Abstract

The composition and function of the human gut microbiome are often associated with health and disease status. Sugar substitute sweeteners are widely used food additives, although many studies using animal models have linked sweetener consumption to gut microbial changes and health issues. Whether sugar substitute sweeteners directly change the human gut microbiome functionality remains largely unknown. In this study, we systematically investigated the responses of five human gut microbiomes to 21 common sugar substitute sweeteners, using an approach combining high-throughput *in vitro* microbiome culturing and metaproteomic analyses to quantify functional changes in different taxa. Hierarchical clustering based on metaproteomic responses of individual microbiomes resulted in two clusters. The noncaloric artificial sweetener (NAS) cluster was composed of NASs and two sugar alcohols with shorter carbon backbones (4 or 5 carbon atoms), and the carbohydrate (CHO) cluster was composed of the remaining sugar alcohols. The metaproteomic functional responses of the CHO cluster were clustered with those of the prebiotics fructooligosaccharides and kestose. The sugar substitute sweeteners in the CHO cluster showed the ability to modulate the metabolism of *Clostridia*. This study provides a comprehensive evaluation of the direct effects of commonly used sugar substitute sweeteners on the functions of the human gut microbiome using a functional metaproteomic approach, improving our understanding of the roles of sugar substitute sweeteners on microbiome-associated human health and disease issues.

**IMPORTANCE** The human gut microbiome is closely related to human health. Sugar substitute sweeteners as commonly used food additives are increasingly consumed and have potential impacts on microbiome functionality. Although many studies have evaluated the effects of a few sweeteners on gut microbiomes using animal models, the direct effect of sugar substitute sweeteners on the human gut microbiome remains largely unknown. Our results revealed that the sweetener-induced metaproteomic responses of individual microbiomes had two major patterns, which were associated with the chemical properties of the sweeteners. This study provided a comprehensive evaluation of the effects of commonly used sugar substitute sweeteners on the human gut microbiome.

## INTRODUCTION

Dietary components, which include carbohydrates (CHO), proteins, fats, minerals, food additives, and other compounds, have been shown to play major roles in shaping the composition and function of the gut microbiome and the associated health consequences (1, 2). Sugar substitute sweeteners are food additives used to increase the sweetness of food while contributing a modest amount of energy, compared to sugar. In the United States, 25% of children and 41% of adults consumed sugar substitute sweeteners, based on data collected from 2009 to 2012 (3), and the popularity of sugar substitute sweeteners has been continuously increasing (4). Sugar substitute sweeteners have been recommended as sugar replacements for better caloric and glycemic control while preserving sweetness (5). Sugar substitute sweeteners can be categorized as noncaloric artificial sweeteners (NASs) of high sweetness intensity, which carry few calories, and sugar alcohols (nutritive sweeteners), which have sweetness comparable to that of sucrose but are indigestible by humans and thus contribute few calories (6).

Despite the proposed health benefits of sugar substitute sweeteners, many studies have found associations between their consumption and the development of diseases and metabolic syndrome (7–9), some of which were initially intended to be prevented by the use of sugar substitute sweeteners. Sweeteners have been found by metagenomics to induce changes in the gut microbiome composition in animals and humans (10–12). Suez et al. demonstrated saccharin (SAC)-induced glucose intolerance in healthy volunteers and showed that these effects were transferable to germfree mice through fecal transplantation (11). However, Serrano et al. found that gut microbial diversity and composition at any taxonomic level, as well as glucose intolerance, were not altered by high-dose SAC supplementation in healthy humans and mice using 16S rRNA sequencing (13). To date, there has been no study that compared the effect of a comprehensive panel of approved sugar substitute sweeteners on the functionality of the *ex vivo* human gut microbiome. Most of the studies were conducted using animal models and focused on only a few sweeteners (11, 14, 15). In addition, comparison across small-scale studies is challenging due to the variation of experimental approaches.

Here, we report a systematic study of the effects of sugar substitute sweeteners on individual human gut microbiomes. Briefly, 21 common sweeteners, covering all sweeteners approved by Health Canada (HC) (16), the U.S. Food and Drug Administration (FDA) (17), and the European Food Safety Authority (EFSA) (18) as food additives, were tested for their impact on the composition and function of five healthy adult microbiomes using the Rapid Assay of Individual Microbiome (RapidAIM) technology (19), consisting of *in vitro* culturing and metaproteomic analysis (see Table S1 in the supplemental material for the 21 sweeteners). To the best of our knowledge, this is the first study using metaproteomic approaches to simultaneously examine the effects of 21 sugar substitute sweeteners on human gut microbiomes. Our results revealed that the sweetener-induced metaproteomic responses of individual microbiomes had two major patterns, which were associated with the chemical properties of the sweeteners.

## RESULTS

### Functional profiles of individual microbiomes were altered by sugar substitute sweeteners.

**FIG 1:**
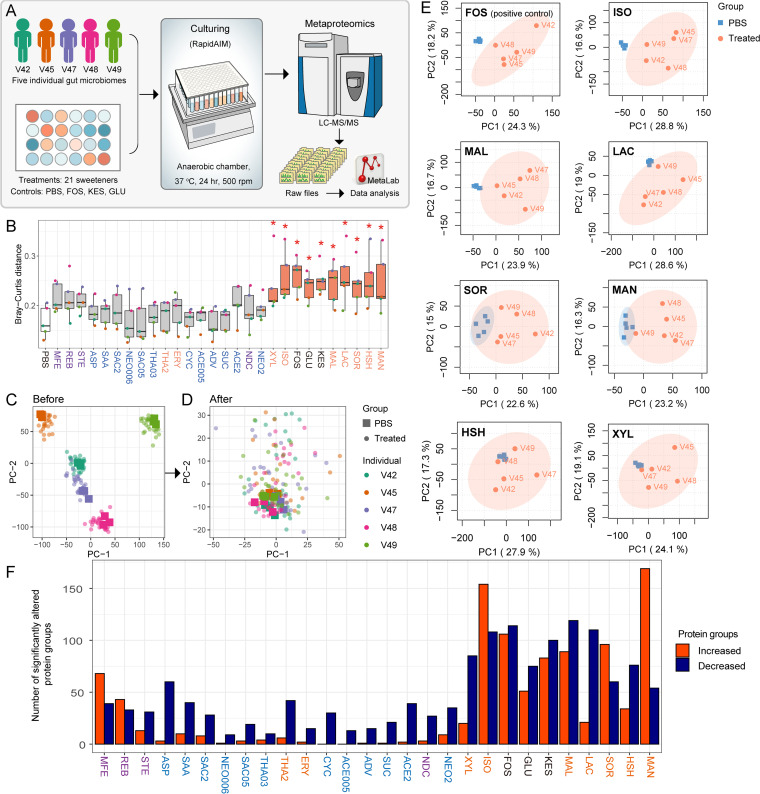
Sweeteners induce metaproteomic changes in the individual microbiomes. Twenty-one sweeteners were analyzed in the study, including four sweeteners tested at two different concentrations (2 mg/mL and cADI). Each sweetener is represented by a three-letter abbreviation. SAC2, NEO2, THA2, and ACE2 correspond to sweeteners at 2 mg/mL, and SAC05, NEO006, THA03, and ACE005 correspond to sweeteners at the cADI (see Table S1 in the supplemental material for abbreviations and specific concentrations). (A) Workflow combining *in vitro* culturing and metaproteomics to study the effects of common sweeteners on the gut microbiome. (B) Bray-Curtis distances of protein group LFQ intensities between sweetener-treated groups and the PBS treated control for each microbiome. Boxes span the interquartile range; jitter colors indicate the microbiome number (same as Fig. 1C and D). *, *P* < 0.05, Wilcoxon rank sum test between each group and the average distance among control sample triplicates. Gray boxes indicate non-significantly altered and orange boxes indicate significantly altered. Colors of sweetener abbreviations are as follows: orange, sugar alcohols; purple, glycoside-type NASs; blue, other NASs; black, controls. The average Bray-Curtis distances of protein group LFQ intensities between PBS triplicates from five individuals were also included (white box). (C) PCA score plot generated from protein group LFQ intensities of all samples. (D) PCA score plot after ComBat transformation to remove interindividual variances. (E) Individual PCA score plots of microbiomes treated with the positive-control FOS and a subset of sweeteners, showing separation from the PBS control (based on data after empirical Bayesian transformation). (F) Numbers of significantly altered proteins under different sweetener treatments. Protein groups with ComBat-normalized intensity fold changes of >2 and *P* values of <0.05 were considered significantly altered. See Table S3 in the supplemental material for the list of significantly altered proteins.

In this study, fecal samples from five healthy volunteers were cultured individually in the presence of each sweetener in an anaerobic workstation for 24 h (Fig. 1A). A total of 197 samples (including quality controls and technical replicates) were analyzed by liquid chromatography-tandem mass spectrometry (LC-MS/MS). The average MS/MS identification rate was 33.8 ± 6.7%. An average of 8,332 ± 1,744 peptides were identified, and 3,428 ± 533 protein groups were quantified from each sample. The Clusters of Orthologous Groups (COG) functional annotation and carbohydrate-active enzyme (CAZyme) annotation of identified protein groups are listed in Table S2 in the supplemental material.

The comparison of Bray-Curtis distances between the sweetener-treated microbiome and nontreated microbiome metaproteomic profiles revealed seven sweeteners (xylitol [XYL], isomalt [ISO], maltitol [MAL], lactitol [LAC], sorbitol [SOR], hydrogenated starch hydrolysates [HSH], and mannitol [MAN]) that significantly altered the metaproteome across all five individual microbiomes (Fig. 1B). Of the eight tested sugar alcohols, only erythritol (ERY) did not show significantly altered metaproteome. Principal-component analysis (PCA) of all samples based on protein group label-free quantification (LFQ) intensities showed, as expected, strong interindividual variations (Fig. 1C); these were due to the nature of the mixture distribution of each protein group among different individuals. We used an empirical Bayesian algorithm (20) to fit each mixture distribution to an empirical distribution to reduce the effect of individual variance on the data set. PCA of the data following transformation showed that the control samples of different individuals now clustered together (Fig. 1D). In agreement, the sweeteners mentioned above and controls showed better separation than other groups (Fig. 1E; also see Fig. S1). In addition, monk fruit extract (MFE)-treated microbiomes showed a distinct cluster away from the phosphate-buffered saline (PBS) group (see Fig. S1). A total of 1,075 protein groups were significantly altered by at least one sweetener (see Table S3). Consistent with Bray-Curtis distances and PCA, a larger number of protein groups were significantly altered by the seven sweeteners (XYL, ISO, MAL, LAC, SOR, HSH, and MAN) mentioned above (Fig. 1F). An average of 54 ± 19 protein groups were identified as CAZyme from each sample. Most of the protein groups were glycoside hydrolases (GHs) (40 ± 16 from each sample). Only 25 of 1,075 significantly altered protein groups were annotated as CAZyme (see Table S3). Two protein groups from GH97 (top protein identification numbers DOM005_GL0010478 and 272559.BF1158), a family composed of α-glucosidase and α-galactosidase, were significantly increased by HSH.

### Sugar substitute sweeteners induce genus-level protein biomass in microbiomes.

We evaluated the effect of sugar substitute sweeteners on total microbial biomass by measuring the total proteins obtained in each sample using a detergent-compatible (DC) protein assay, as described in Materials and Methods. For most sweeteners, their effects on the individual microbiomes varied, while ISO and thaumatin at 2 mg/mL (THA2) increased the total biomass in all five microbiomes (see Fig. S2).

**FIG 2:**
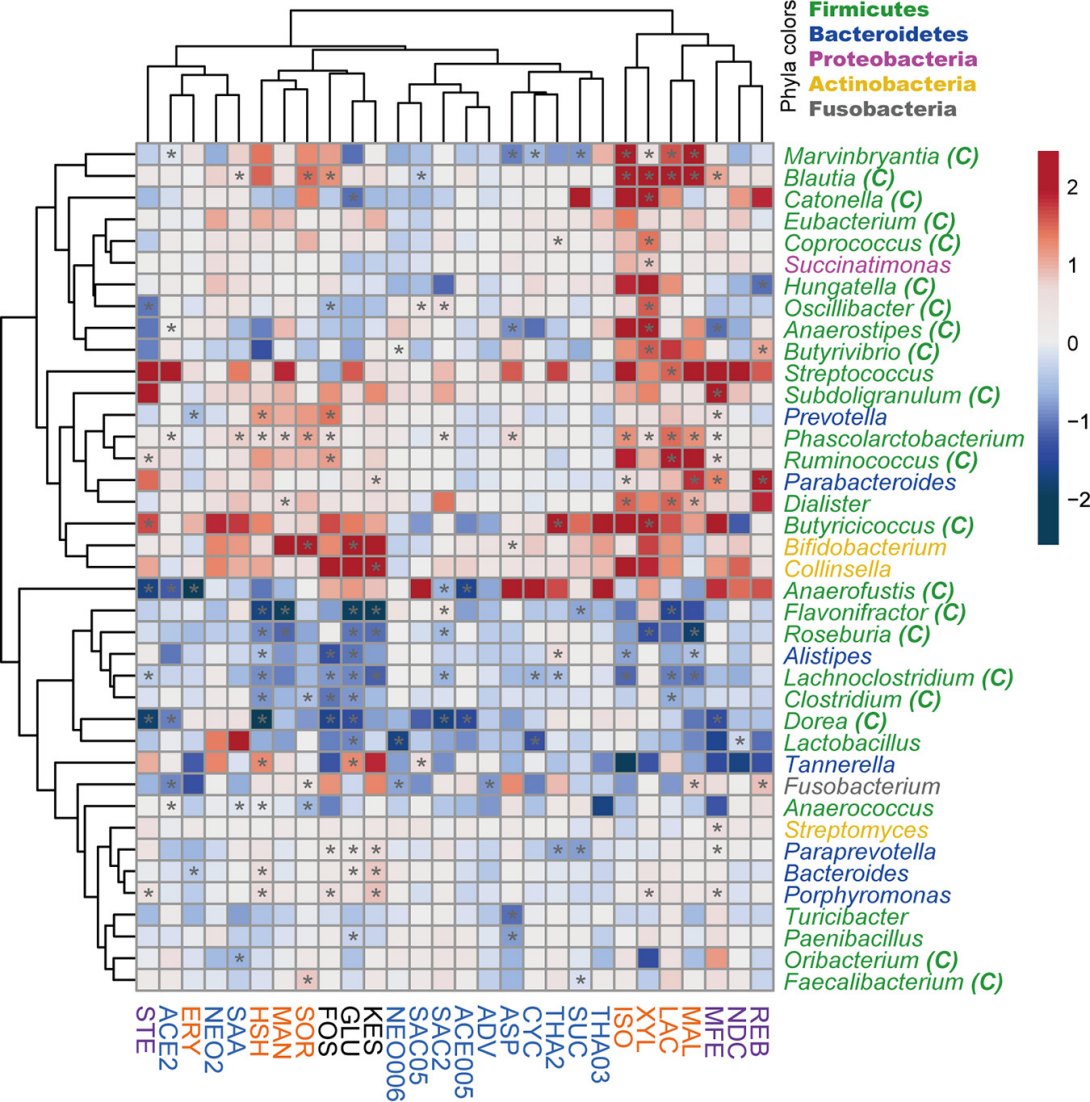
Sweeteners induced gut microbiome genus-level protein abundance changes. Sweeteners are named as in Fig. 1. The heatmap shows log_2_ fold changes in genus-level protein abundance of sweetener-treated samples versus the PBS control. For each treatment, the averaged genus-level protein abundance of all five microbiomes was used for coloring and clustering. *, *P* < 0.05, Wilcoxon rank sum test. Genera that were detected in PBS controls in at least four of the five microbiomes are shown. Genera from *Clostridia* are indicated with C in parentheses.

Genus-level protein abundance was then calculated from the distinctive peptide intensities of each genus measured by LC-MS/MS and the total microbial protein biomass of each sample. Most sweeteners showed significant effects on the abundance of at least one genus. Clustering based on the effects of genus-level biomass shows that the genus-level protein biomass composition was affected in patterns by different classes of sugar substitute sweeteners (Fig. 2). Glycoside-type NASs rebaudioside A (REB), neohesperidin dihydrochalcone (NDC), and MFE, as well as sugar alcohols MAL, LAC, XYL, and ISO, clustered together and formed a distinct cluster from other sweeteners and controls. This cluster showed significant increases in several genera, such as *Dialister*, *Parabacteroides*, *Ruminococcus*, *Phascolarctobacterium*, *Butyrivibrio*, *Blautia*, and *Marvinbryantia*. Sugar alcohols SOR, MAN, and HSH clustered with controls fructooligosaccharide (FOS), kestose (KES), and glucose. This cluster featured significant increases in *Actinobacteria* genera *Bifidobacterium* and *Collinsella* and decreases in *Dorea*, *Clostridium*, *Lachnoclostridium*, *Alistipes*, *Roseburia*, and *Flavonifractor*. Genera *Coprococcus*, *Oscillibater*, *Anaerostipes*, and *Butyrivibrio* were increased by XYL but not by any other sugar alcohols.

### Functional metaproteomic analysis segregates the sugar substitute sweeteners into two groups.

**FIG 3:**
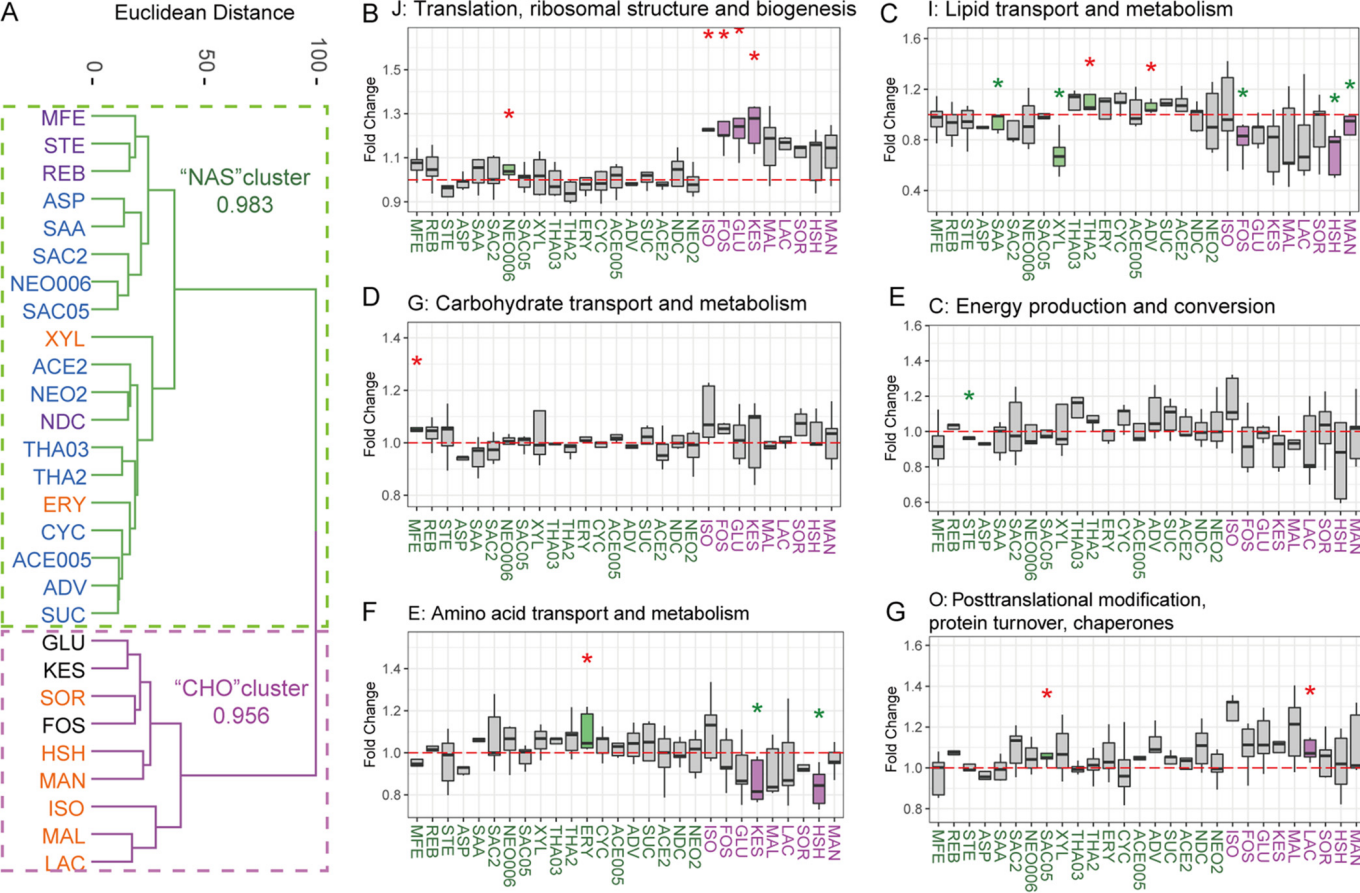
Sweeteners induced functional changes in the gut microbiome. Sweeteners are named as in Fig. 1. (A) Clustering of sweeteners based on induced functional responses. Euclidean distances between sweeteners were calculated based on averaged log_2_ fold changes of COG abundances of sweetener-treated samples versus the PBS-treated control. Bootstrapping scores of the two major clusters are shown. (B to G) Fold changes between the treated group and the PBS-treated control for several COG categories. Colored boxes indicate significantly changed COG categories. Red and green asterisks indicate significant increases and decreases, respectively. *, *P* < 0.05, Wilcoxon rank sum test. Responses of all other COG categories are shown in Fig. S3 in the supplemental material.

Of the identified protein groups, 93.5% had COG functional annotation. Sweeteners were categorized into two major clusters using COG abundances (Fig. 3A; also see Fig. S3). Bootstrapping of the two major clusters gave scores of 0.983 and 0.956, respectively, indicating high clustering robustness (Fig. 3A). We named the two clusters the NAS and CHO clusters according to the properties of the sweeteners. Statistical analysis at the COG category level identified 14 of 21 COG categories that were significantly altered by at least two compounds (Fig. 3B to G; also see Fig. S3).

In the NAS cluster, all NASs were included, plus sugar alcohols ERY and XYL, which have shorter carbon backbones (see Fig. S4). Interestingly, although four sugar alcohols, MAL, lactitol monohydrate, XYL, and ISO, were clustered with three of the NASs, i.e., REB, NDC, and MFE, in the taxonomic analysis in Fig. 2, the functional responses of the microbiome to these two clusters were different. While XYL still belonged to the NAS cluster, the three other sugar alcohols (i.e., MAL, lactitol monohydrate, and ISO) belonged to the CHO cluster. XYL showed marked effects on the metaproteome, including significantly decreased lipid transport and metabolism and cell motility and significantly increased coenzyme transport and metabolism (Fig. 3C; also see Fig. S3). Extracellular structures were significantly promoted by the MFE-stevioside (STE)-REB subcluster (see Fig. S3). The CHO cluster included all remaining sugar alcohols and positive controls, all of which are CHOs. In the CHO cluster, sugar alcohols SOR, MAN, LAC, MAL, ISO, and HSH cannot be digested by the human body. Therefore, despite incomplete absorption of sugar alcohols in the small intestine (21), they can reach the colon intact, serving as substrates for microbial fermentation to produce hydrogen gas, carbon dioxide, methane, and short-chain fatty acids (SCFAs) (22). Compounds in this cluster were found to induce marked responses in the metaproteomes in a similar pattern, including an increase in translation, ribosomal structure, and biogenesis and a decrease in lipid transport and metabolism (Fig. 3B and C). In addition, proteins with general function prediction only (COG category R) were significantly increased by a subset of the CHO cluster (see Fig. S3). Sugar alcohols have been shown to induce gastrointestinal symptoms, including bloating, laxative effects, and abdominal pain (23). Accordingly, we showed that some sugar alcohols, such as ISO, significantly reduced cell motility, which has been reported to be associated with increased susceptibility to intestinal expulsion and larger fluctuation in absolute abundance (24).

### CHO cluster sweeteners modulated the metabolism of *Clostridia*.

**FIG 4:**
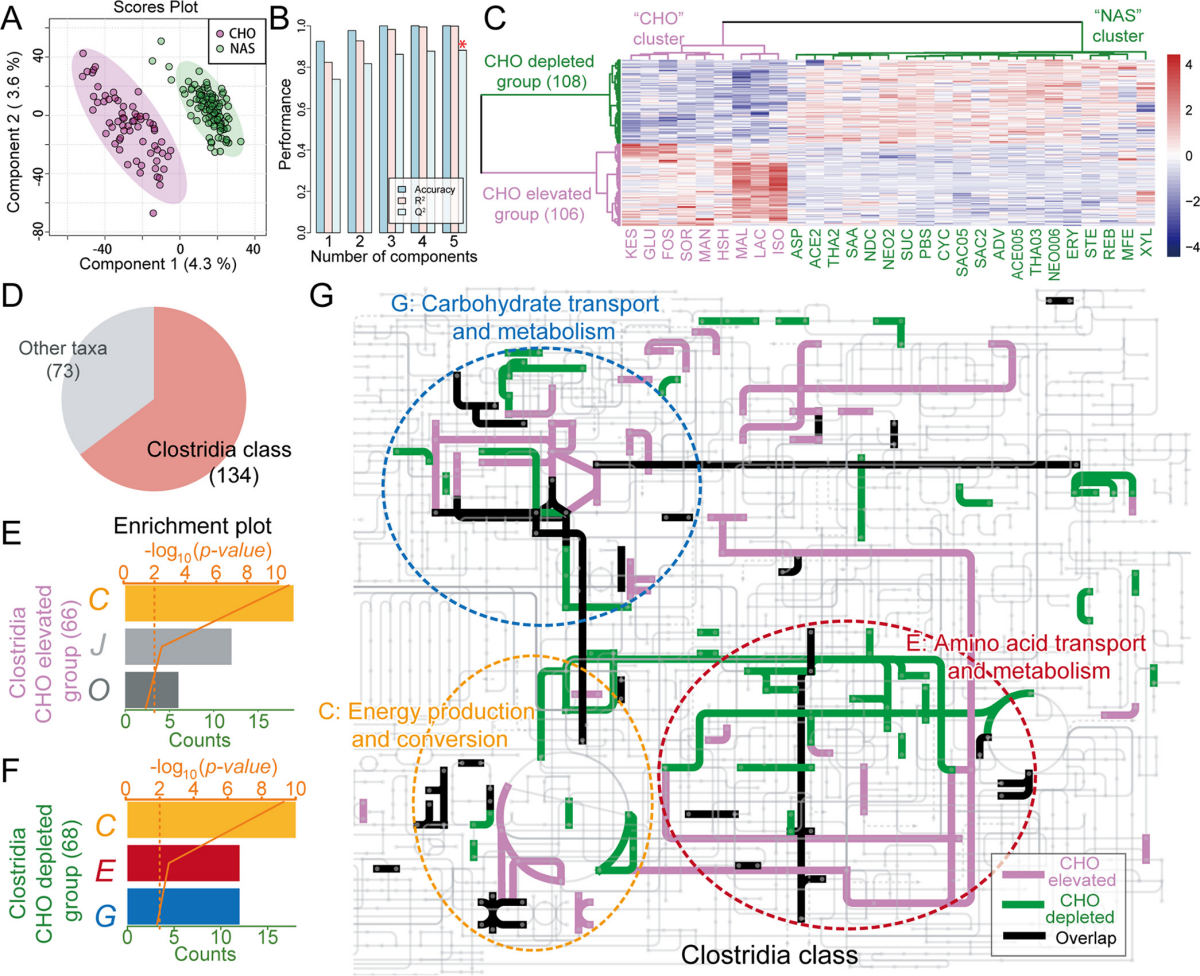
Taxonomic and functional profiles of discriminative proteins for the CHO cluster and NAS cluster revealed by PLS-DA. (A) PLS-DA score plot for differential protein profiles across the CHO cluster and NAS cluster sweeteners. (B) PLS-DA cross-validation results. (C) Heatmap of the intensity of 214 discriminative proteins under treatment with different sweeteners (sweeteners are named as in Fig. 1). (D) Taxonomic sources of discriminative proteins. (E and F) Enriched COG categories of discriminative proteins in *Clostridia* in the CHO elevated group (E) and the CHO depleted group (F). (G) Pathways of discriminative proteins in *Clostridia* in the CHO elevated group and the CHO depleted group. Proteins related to COG categories C, G, and E are framed in the dashed circles.

Partial least-squares discriminant analysis (PLS-DA) was performed to identify the most important differences in functional effects between the CHO cluster and the NAS cluster (Fig. 4A and B). A total of 214 of 3,608 protein groups had a variable importance in projection (VIP) score of >2 in the first five components, which indicates that these proteins explain the most important differences in functional profiles between the two clusters. These proteins were referred to as discriminative proteins. The intensity profiles of these 214 discriminative proteins in response to the treatment with different sweeteners were segregated into two well-defined groups (Fig. 4C). A total of 106 of the discriminative proteins that had greater abundance in the CHO cluster were referred to as the CHO elevated group. Sweeteners of the NAS cluster were clustered with the PBS group, indicating that proteins enriched in the NAS cluster were decreased in the CHO cluster. Therefore, 108 of the discriminative proteins that had greater abundance in the NAS cluster were referred as the CHO depleted group.

Because not all of the identified peptides had taxonomic information from the MetaLab, 207 of the 214 discriminative proteins were assigned taxonomic information from peptides, as described in Materials and Methods, and 62.6% of the discriminative proteins (134 proteins) were from class *Clostridia* (Fig. 4D). Forty-six of the 134 proteins could be assigned to the genus level, as they were from 11 genera, i.e., *Roseburia* (21 proteins), *Clostridium* (6 proteins), *Blautia* (4 proteins), *Eubacterium* (4 proteins), *Lachnoclostridium* (3 proteins), *Ruminococcus* (3 proteins), *Anaerostipes* (1 protein), *Coprococcus* (1 protein), *Faecalibacterium* (1 protein), *Fusicatenibacter* (1 protein), and *Oscillibacter* (1 protein). Functional enrichment analysis of each group of discriminative proteins from *Clostridia* was performed. The CHO elevated group was enriched in COG categories C (energy production and conversion), J (translation), and O (posttranslational modification, protein turnover, and chaperone functions), while the CHO depleted group was enriched in COG categories C, E (amino acid metabolism and transport), and G (CHO metabolism and transport) (Fig. 4E and F). Although both the CHO increased group and the CHO depleted group from *Clostridia* were enriched in COG category C, the enriched proteins from the two groups had different functions (see Table S4). Pathway analysis also showed that proteins from the two groups were from different metabolic pathways (Fig. 4G). In particular, iron-dependent proteins that are involved in the tricarboxylic acid (TCA) cycle between succinyl-coenzyme A (CoA) and malate and its linkage to oxidative phosphorylation (COG0427, COG0479, COG1145, COG1838, COG1951, and COG2048) were highly enriched. The CHO cluster sweeteners had effects on the protein abundance of discriminative proteins similar to those of FOS and KES, which are commonly used prebiotics.

## DISCUSSION

Although there have been studies of the effects of sweeteners on the human gut microbiome (11, 12), a comparison of the effects of a large number of different sugar substitute sweeteners on human gut microbes has not been reported. In this study, we investigated the taxonomic and functional responses of five individuals’ human gut microbiomes cultured *in vitro* to 21 common sugar substitute sweeteners. Among the 21 sweeteners, 13 are NASs, with diverse chemical properties and high sweetness intensities, and 8 are sugar alcohols, which are CHOs with low digestibility (23). We used a previously developed *in vitro* model that was shown to maintain microbial taxon and function (19, 25). We tried to use this *in vitro* model to reveal the real functional change in the human gut microbiome. This *in vitro* model has been shown to recapitulate microbiome changes observed *in vivo* (25). The purpose of the *in vitro* model is to better understand the direct effects of compounds on individual microbiomes and, as with any assays, would need *in vivo* confirmation.

We observed that seven sugar substitute sweeteners (XYL, ISO, MAL, LAC, SOR, HSH, and MAN) significantly altered the metaproteome across the five gut microbiomes (Fig. 1B). The remaining sweeteners had no global metaproteome effects. Although MFE showed a clear separation between control and treatment by PCA, the alteration was not significant using Bray-Curtis distance. A previous study reported that SAC alters the gut microbiota and induces glucose intolerance in a mouse model (11). While we did not observe any global effects of SAC on the metaproteome using our *in vitro* assay (Fig. 1B), we observed changes at the individual level, indicating an individual-specific response to SAC (see Fig. S1 in the supplemental material). A similar individual-specific response was observed for sucralose (SUC), which has been shown by others to alter the composition of the gut microbiota in a rat model (14, 15). From the PCA plots, we observed that, compared with PBS controls, there were larger variations between replicates with the treatment with NAS cluster sweeteners. This suggests interindividual differences in the response of the NAS cluster sweeteners. It is noted that only one or two concentrations of each sweetener were tested in this study. The number of significantly altered protein groups with SAC2 was larger than that with SAC05. The same situation happened between THA2 and THA03, neotame at 2 mg/mL (NEO2) and NEO006, and acesulfame at 2 mg/mL (ACE2) and ACE005 (Fig. 1F), which indicated that higher concentrations of sweeteners could likely have a larger impact on the human gut microbiome. The impacts of different concentrations of sweeteners need further investigation. In addition, most sugar substitute sweeteners had limited effects on the biomass of the microbiome. Only ISO and THA2 led to a biomass increase in all five microbiomes (see Fig. S2). Therefore, sugar substitute sweeteners that affect the microbiome mostly do not affect the protein biomass but instead have systemic or individual effects on taxonomic balance and expressed proteins and enzymes.

A total of 1,075 protein groups were significantly altered by one or more of the sugar substitute sweeteners (see Table S3). Although it is not possible to assess the possible functional impact of the changes in each protein group, some examples are noteworthy. For example, an average of 54 CAZymes, an important group of enzymes for microbiome function, were identified in each sample, a level consistent with a previous study (26). Most of the identified CAZymes were GHs, which can account for one-half of the classified CAZmes (27). A protein group from GH133 (top protein identification number V1.CD38-0_GL0163641) was reduced by 13 of the sugar substitute sweeteners. GH133 CAZymes are glycogen-debranching enzymes (α-1,6-glucosidases). The decrease in this enzyme class likely indicates that the sugar substitute sweeteners depressed the release of glucose from glycogen storage by some bacteria in the microbiome.

There were also specific groups of proteins that were affected by multiple sugar substitute sweeteners. For example, the protein group (top protein identification number MH0148_GL0062639) classified as outer membrane protein (OMP) OmpA from the genus *Alistipes* was decreased by four sugar substitute sweeteners. OmpA is one of the most abundant OMPs (28) and is involved in many processes, particularly responses to stresses. It has been shown in some species to provide resistance to antibiotics (29). Its decrease by the four specific sugar substitute sweeteners could impact its roles in *Alistipes*.

The genus-level taxonomic changes of the gut microbiome with different sugar substitute sweeteners were evaluated. A sweetener cluster consisting of both glycoside-type NASs (REB, NDC, and MFE) and sugar alcohols (MAL, LAC, XYL, and ISO) significantly increased protein abundance from several genera in *Firmicutes*. A single-species-based study revealed that XYL was largely utilized by Anaerostipes caccae from *Firmicutes* to produce butyrate and promoted the growth of the species (30). The effects of sweeteners on other genera from *Firmicutes* need to be studied further. In addition, controls FOS, KES, and glucose were clustered with sugar alcohols SOR, MAN, and HSH. It is noted that this clustering result was different from the functional profile clustering. While metaproteomics measure the abundances of different taxa by summarizing the overall protein intensities, functional profiles provide a deeper layer of information by comparing functional proteins in the community and are more relevant to the actual functionality and state of the microbiomes.

By analyzing the functional profiles of the cultured microbiomes, we segregated all of the sweeteners into two clusters, NAS and CHO. To further investigate the different effects of the two clusters of sweeteners on the human gut microbiome, discriminative proteins with PLS-DA VIP scores of >2 were identified. FOS and KES clustered with the CHO cluster, which indicated that the CHO cluster might have functional effects similar to those of FOS and KES. Most of the discriminative proteins were from *Clostridia*. *Clostridia* accounts for at least 10 to 40% of the total bacteria in gut microbiota (31), and members from this class have significant potential as probiotics (32).

Functional enrichment analysis of discriminative proteins from *Clostridia* revealed that proteins with greater abundances in the CHO cluster (CHO elevated group) and proteins with greater abundances in the NAS cluster (CHO depleted group) were both enriched in COG category C. Enriched proteins from the two groups corresponded to different functions (see Table S4), with differences in specific pathways, as shown by the metabolic pathway analysis (Fig. 4G). In particular, the enrichment of iron-dependent pathways in the TCA cycle and oxidative phosphorylation indicated increased cellular energy metabolism in *Clostridia* in response to the CHO cluster. Other proteins from the two groups also corresponded to different pathways, indicating that the two clusters of sweeteners had effects on different metabolic pathways of *Clostridia*. In addition, the CHO elevated group was enriched in COG function categories J (translation, ribosomal structure, and biogenesis) and O (posttranslational modification, protein turnover, and chaperone functions). The greater abundance of proteins from these two categories indicated that the CHO cluster sweeteners might promote the cellular growth of *Clostridia*. A previous study showed that the traditional prebiotic FOS and probiotic *Lactobacillus* can increase the proportion of *Clostridia* in gut microbiomes (33), and CHO cluster sweeteners might have similar potential as these prebiotics and probiotics. Discriminative proteins with greater abundance in the NAS cluster (CHO depleted group) were enriched in COG categories C, G, and E (amino acid transport and metabolism). The functional annotation of enriched proteins (see Table S4) showed that certain numbers of these discriminative proteins in COG categories C (5 of 18 proteins) and G (4 of 12 proteins) were related to glycerol transport and alcohol metabolism. This suggested inhibition of the glycerol transport and alcohol metabolic pathways by the CHO cluster.

In conclusion, we examined the effects of 21 sweeteners on the *ex vivo* human gut microbiomes from five individuals. Seven sweeteners significantly altered the metaproteomes across the five microbiomes. Functional profiles of the microbiomes clustered all of the sweeteners into the NAS cluster and the CHO cluster. Prebiotics FOS and KES clustered with the CHO cluster. Most discriminative proteins for the two clusters were from *Clostridia*. Functional enrichment analysis and pathway analysis revealed that the two sweetener clusters had effects on different pathways of *Clostridia*. CHO cluster sweeteners had effects similar to those of the prebiotics FOS and KES. Our study revealed the functional effects of sweeteners on the human microbiome and suggested the prebiotic potential of the sugar alcohol sweeteners.

## MATERIALS AND METHODS

### Sugar substitute sweeteners and determination of concentrations.

The concentrations of the sugar substitute sweeteners used in the assay were determined based on their consumption levels in the general public, the acceptable daily intake (ADI), and the proportion of consumed sweeteners that could reach the colon (see Fig. S5 in the supplemental material). Twelve of the tested sweeteners had ADI data defined by FDA or EFSA. The culturing concentration that met the ADI (cADI) for each sweetener was calculated based on consumption from an average participant body weight of 70.3 kg, normalized to 200 g of colon content. Calculation of the cADI was conducted based on the following formula:

(1) $$\begin{array}{l} \text{cADI}=\frac{\text{ADI}\left(\text{mg}\;\text{k}{\text{g}}^{-1}\;{\text{day}}^{-1}\right)\times \text{average volunteers body weight}\;70.3\;\text{kg}}{{\left(\text{weight of culture system}\;1\;\text{g}\right)}^{-1}\times \text{weight of colon content}\;200\;\text{g}}\\ \;\times \;1\;\text{m}{\text{L}}^{-1}\times {\text{MW}}^{-1}\times \text{proportion that reaches the colon}\;(\text{\%}) \end{array}$$

Sweeteners with cADI values much lower than 2 mg/mL were tested both at the cADI and at 2 mg/mL to facilitate the comparison of their effects on the microbiome with those of other sweeteners. For sweeteners without defined ADI, in the case of all sugar alcohols the concentration used was standardized to 2 mg/mL, which would represent consumption levels lower than those in the general public (34–36). Since the cADI of advantame exceeded its solubility and the ADI would represent a consumption level much higher than that in the general public (37), advantame was tested at 2 mg/mL, about one-fifth of the cADI. In addition, aspartame (ASP), THA, and salt of aspartame-acesulfame (SAA) are known to be completely metabolized before reaching the colon (38, 39), resulting in cADI values of 0. A study has shown that CHOs that can be absorbed by the small intestine can still enter the large intestine and be fermented by the colonic microbiota (40). We included ASP, THA, and SAA in this study, assuming that a portion of these sweeteners may also reach the colon.

### Human stool sample collection and culturing.

The study was approved by the Ottawa Health Science Network Research Ethics Board at the Ottawa Hospital (protocol number 20160585-01 H). Informed consent was obtained from all subjects involved in the study. Stool samples from five healthy volunteers (23 to 48 years of age; 3 males and 2 females) were included in this study. Exclusion criteria included the diagnosis of irritable bowel syndrome (IBS), inflammatory bowel disease (IBD), or diabetes mellitus; antibiotic use or gastroenteritis episode in the past 3 months; use of probiotic/prebiotic, laxative, or antidiarrheal drugs in the past month; or pregnancy. Volunteers included in this study were self-assessed as non-sweetener consumers, based on their food and nutritional supplement consumption. The stool samples were collected on site and immediately transferred into an anaerobic workstation (5% H_2_, 5% CO_2_, and 90% N_2_, at 37°C). A 20% (wt/vol) stool slurry was made in sterile prereduced PBS (pH 7.6) containing 10% (vol/vol) glycerol and 1 g/L l-cysteine, vortex homogenized, and filtered through gauze. The filtered slurry aliquots were stored at −80°C until culturing.

The frozen fecal samples were thawed at 37°C and immediately transferred into the anaerobic workstation, vortex homogenized, and inoculated at 2% (wt/vol) into 96-deep-well plates containing prereduced, optimized microbiome culture medium (19, 25) and a sweetener (the manufacturers and concentrations used are shown in Table S1 in the supplemental material), a positive control (2 mg/mL FOS or 2 mg/mL KES), or the negative control (PBS). The culture medium was composed of 2.0 g/L peptone water, 2.0 g/L yeast extract, 0.5 g/L l-cysteine hydrochloride, 2 mL/L Tween 80, 5 mg/L hemin, 10 μL/L vitamin K_1_, 1.0 g/L NaCl, 0.4 g/L K_2_HPO_4_, 0.4 g/L KH_2_PO_4_, 0.1 g/L MgSO_4_·7H_2_O, 0.1 g/L CaCl_2_·2H_2_O, 4.0 g/L NaHCO_3_, 4.0 g/L porcine gastric mucin, 0.25 g/L sodium cholate, and 0.25 g/L sodium chenodeoxycholate. The plates were shaken at 500 rpm on shakers (MS3; IKA, Germany) in the anaerobic workstation at 37°C for 24 h. Following anaerobic culture, samples were processed to isolate bacterial pellets as described previously (19). Bacterial pellets were stored at −80°C until LC-MS/MS sample preparation.

### Metaproteomic sample preparation.

Proteins were extracted from bacterial pellets according to the method described by Li et al. (19). Briefly, bacterial pellets were resuspended in bacterial lysis buffer containing 4% (wt/vol) sodium dodecyl sulfate (SDS), 8 M urea, 50 mM Tris-HCl (pH 8.0), cOmplete mini protease inhibitor tablets, and PhosSTOP inhibitor (MilliporeSigma). Samples were sonicated (Qsonica, USA) at 8°C, at 50% amplitude, for 10 min with a 10 s on/10 s off working cycle. Lysates were centrifuged at 16,000 × *g* at 8°C for 10 min to remove cell debris. Supernatant protein concentrations were measured with the Bio-Rad DC protein assay reagent in triplicate and were used to calculate the total biomass of the microbiome after culturing.

Proteins from each sample were precipitated overnight at −20°C by mixing lysis supernatant with a protein precipitation buffer containing 50% (vol/vol) acetone, 50% (vol/vol) ethanol, and 0.1% (vol/vol) acetic acid, at 1:5 ratio (vol/vol). Precipitated proteins were collected by centrifugation at 16,000 × *g* at 4°C for 25 min. Proteins were washed three times with 1 mL of −20°C acetone and pelleted by centrifugation at 16,000 × *g* at 4°C for 25 min. Following acetone washes, proteins were dissolved in 6 M urea-50 mM ammonium bicarbonate (ABC) (pH 8.0). Protein concentrations were determined as described above. Protein aliquots (50 μg) were reduced with 10 mM dithiothreitol at 56°C for 30 min, with shaking. Protein alkylation was then performed with 20 mM iodoacetamide in the dark for 40 min at room temperature. Samples were diluted 10-fold with 50 mM ABC (pH 8.0). Trypsin (Worthington Biochemical Corp., Lakewood, NJ) was added to a trypsin/protein mass ratio of 1:50, and digestion was performed at 37°C for 19 h at 850 rpm. Trypsin digestion was stopped by adding 50 μL 10% (vol/vol) formic acid to a final pH of 2 to 3. Desalting was performed on in-house-made 96-channel filter tip plates packed with 5 mg 10-μm C_18_ resin (Dr. Maisch GmbH, Ammerbuch, Germany). Desalted samples were freeze-dried and stored at −20°C.

### LC-MS/MS analysis.

An Eksigent NanoLC system (nano2D ultra) coupled with a Q Exactive mass spectrometer (Thermo Fisher Scientific Inc.) was used for analysis. Tryptic peptides were reconstituted in 0.1% (vol/vol) formic acid to approximately 0.25 μg/μL, and 1 μg of peptides was loaded. The column used for peptide separation was of 75-μm inner diameter and was 15 cm long, packed with reverse-phase C_18_ resin (1.9-μm/120-Å ReproSil-Pur C_18_ resin; Dr. Maisch GmbH). A 90-min gradient with acetonitrile changing from 5% to 30% (vol/vol) was used, at a flow rate of 300 nL/min. Solvent A was composed of 0.1% (vol/vol) formic acid, and solvent B was composed of 0.1% (vol/vol) formic acid and 80% (vol/vol) acetonitrile. MS analysis was performed with a Q Exactive mass spectrometer (Thermo Fisher Scientific Inc.). Full MS scans were performed from *m/z* 300 to *m/z* 1,800, and data-dependent MS/MS scans were performed for the 12 most intense ions. MS and MS/MS scans were performed with resolutions of 70,000 and 17,500, respectively. Samples were loaded in a randomized order. In this study, 197 samples were analyzed over a period of 23 days. All raw data from LC-MS/MS have been deposited with the ProteomeXchange Consortium (http://www.proteomexchange.org) via the PRIDE (41) partner repository via the data set identifier PXD030458.

### Protein identification, quantification, and profiles.

The MetaLab software (version 1.2.0) was used for peptide/protein identification and quantification, peptide taxonomic assignment, and protein functional annotation (42). The searches were performed against a database based on the integrated gene catalog (IGC), which included close-to-complete sets of genes for most gut microbes (43). MaxQuant is used as the search engine in the MetaLab workflow for peptide/protein identification (44). Carbamidomethyl (C) was set as a fixed modification, and protein N-terminal acetylation (protein N-term) and oxidation (M) were set as variable modifications. The enzyme was set as trypsin, and two missed cleavages were allowed. The false-discovery rate (FDR) was set as 0.01 for both the peptide and protein levels. Because one peptide can be matched to multiple proteins, the database search engine adopts the protein inference method to determine the attribution of peptides (45). Basically, two proteins join in a protein group when one of the proteins has a set of peptides equal to or completely contained in the other protein. Shared peptides remain in all groups where they occur, but they are most parsimoniously associated with the group that has the highest number of identified peptides (“razor” peptides). Only unique and razor peptides are used for protein quantification. The taxonomic assignment of peptides can be found below in “Microbial taxonomic analysis.”

Analysis of changes in the gut metaproteome was based on the quantified protein groups. LFQ intensities of each protein group were first normalized by the estimated size factor calculated using the R package DESeq2 (46). Bray-Curtis distances between samples were calculated based on the normalized intensities using the R package vegan (47). For PCA, protein groups were filtered based on the criteria that the protein group appears in at least one treatment in at least three of the five tested microbiomes (60%). The intensities were then log_10_ transformed, and PCA was performed using the R package stats. To reduce the effect of interindividual microbiome variation on data analysis, the distribution of each protein group among individual microbiomes was fitted to the same empirical distribution using an empirical Bayesian algorithm with ComBat (20) on iMetaLab (48). ComBat was also used for the intensities before log_10_ transform. ComBat-normalized intensities for each protein group were added to the absolute value of the smallest intensity of the protein group to avoid negative numbers. The intensities were then uploaded to the differential protein analyzer on iMetaLab with the default parameters. Under different treatments, protein groups with intensity fold changes of >2 and *P* values of <0.05 were considered significantly altered protein groups.

The CAZyme profile of the microbiome was acquired from the major protein of protein groups. The protein sequences of major proteins were extracted from the IGC database and then annotated with dbCAN meta server with two tools, HMMER (E value of <1*e*−15, with coverage of >0.35) and DIAMOND (E value of <1*e*−102) (49).

### Microbial taxonomic analysis.

Taxonomic assignment of each peptide was performed based on the lowest common ancestor (LCA) algorithm. If one peptide is identified in more than one taxon, then the peptide is assigned to the LCA of those taxa. Using a previously constructed pep2tax database integrated with the MetaLab software (version 1.2.0), each identified peptide was assigned to a taxon (42). The abundance of each taxon was calculated by summarizing the intensities of all distinctive peptides assigned to that taxon. The relative abundance of taxa in a specific taxonomic rank was calculated by normalization to the summed abundance of all taxa at that rank. For comparison of absolute taxon abundance between samples, relative abundances were multiplied by the total microbial biomass calculated using protein concentration data. Fold changes were calculated between sweetener-treated samples and PBS-treated control samples from the same microbiome.

### Microbial function analysis.

Functional annotation was carried out in the MetaLab software, and each identified protein group was assigned to a COG category. The relative abundance of each COG was calculated based on the summed LFQ intensities of all assigned protein groups. Clustering of sweeteners was based on the fold change of relative COG intensities, averaged across all tested sweeteners. The Euclidean distance between sweeteners was calculated, and the clustering was performed with the ward.D method, using the R package stats. Bootstrapping evaluation (50) of the two major clusters was performed using the R package fpc (51), with the number of resampling runs being 100.

PLS-DA was performed to identify discriminative proteins that reveal the difference in the effects of the two clusters of sweeteners. PLS-DA was performed in MetaboAnalyst 5.0 (52), and discriminative proteins with VIP scores of >2 were selected. The taxonomic sources of the discriminative proteins were obtained by the method described in a previous study (53). Briefly, among all of the peptides contained in the discriminative protein, the taxonomic source of the peptide with the most detailed taxonomic information was regarded as the taxonomic source of the protein. All of the peptides from a discriminative protein were acquired from the protein group table from the MetaLab output, and the taxonomic information of the peptides was obtained through the taxonomy table from MetaLab. Functional enrichment analysis of these discriminative proteins was performed using an online enrichment analysis tool (https://shiny.imetalab.ca), and the *P* value threshold was set at <0.05. Visualization of pathways was performed using COG accession numbers in iPath3 (54).

### Data availability.

All raw data from the LC-MS/MS analyses have been deposited with the ProteomeXchange Consortium (http://www.proteomexchange.org) via the PRIDE partner repository (PXD030458).

## Supplementary Material

Reviewer comments
