# Supplementary material for: Comprehensive Assessment of Functional Effects of Commonly Used Sugar Substitute Sweeteners on Ex Vivo Human Gut Microbiome
Source: Microbiol Spectr. 2022 Jun 13;10(4):e00412-22. doi: 10.1128/spectrum.00412-22 (PMC9431030; doi:10.1128/spectrum.00412-22)
Supplement: Supplemental file 1 — Supplemental material. Download spectrum.00412-22-s0001.pdf, PDF file, 1.4 MB [file spectrum.00412-22-s0001.pdf]

Comprehensive assessment of functional effects of commonly used sugar substitute sweeteners on *ex vivo* human gut microbiome

Supplementary Figures

Supplementary Figure S1. Individual PCA plots of other sweeteners, positive control KES and dietary sugar control GLU.

Supplementary Figure S2. Fold change of total protein amount obtained for each treatment group compared to PBS control.

Supplementary Figure S3. Responses of other COG categories in addition to Figure 3.

Supplementary Figure S4. Structure of sweeteners.

Supplementary Figure S5. Procedures used to determine sweetener concentrations.

Supplementary Tables

Supplementary Table S1 Summary of sweeteners.

Supplementary Table S2 Functional annotation of the top protein of the identified protein groups. (See .xlsx supplemental file)

Supplementary Table S3 Change of ComBat normalized protein group intensities under different treatment of sweeteners. (See .xlsx supplemental file)

Supplementary Table S4 Functional annotation of enriched discriminative proteins from Clostridia. (See .xlsx supplemental file)

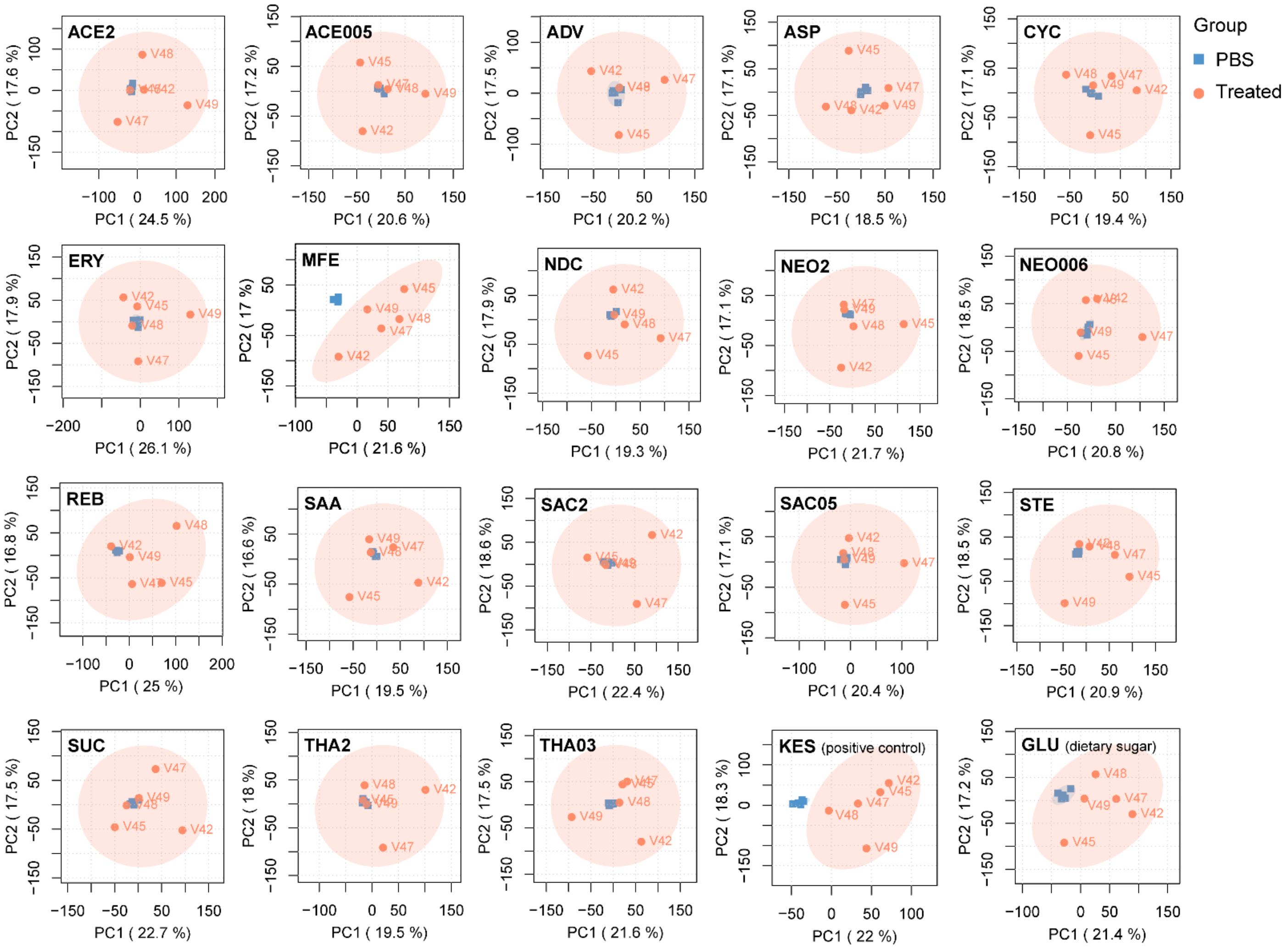

Supplementary Figure S1. Individual PCA plots of other sweeteners, positive control KES and dietary sugar control GLU.

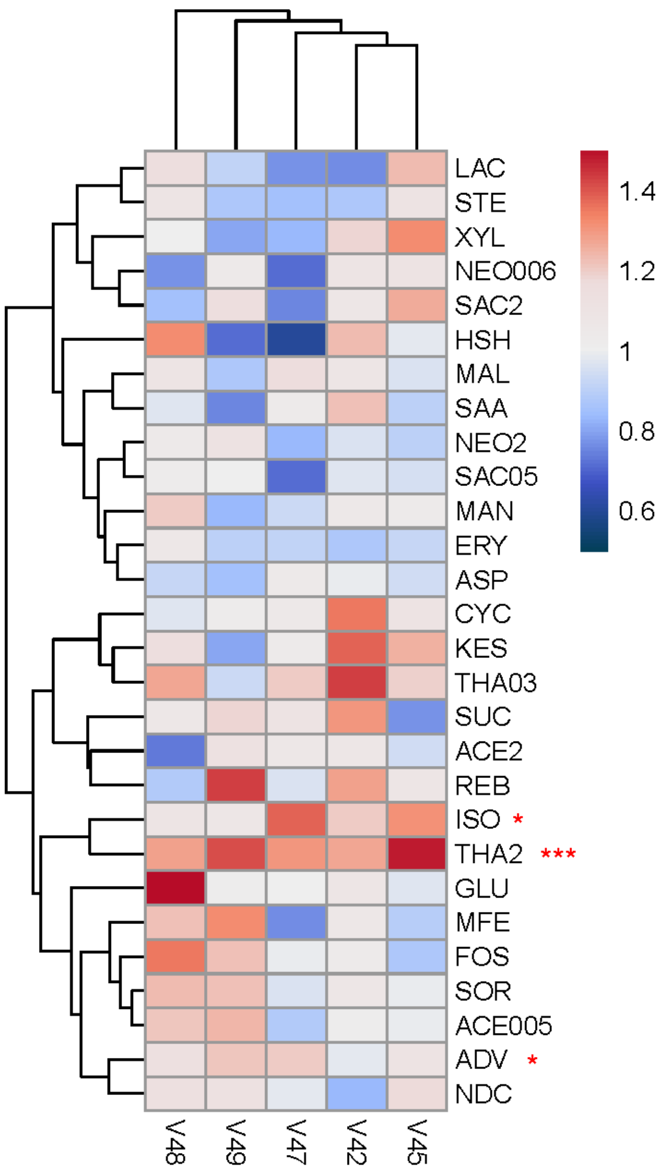

Supplementary Figure S2. Fold change of total protein amount obtained for each treatment group compared to PBS control. * and *** denotes $p < 0.001$ and $p < 0.05$, respectively, by two-sided t-test.

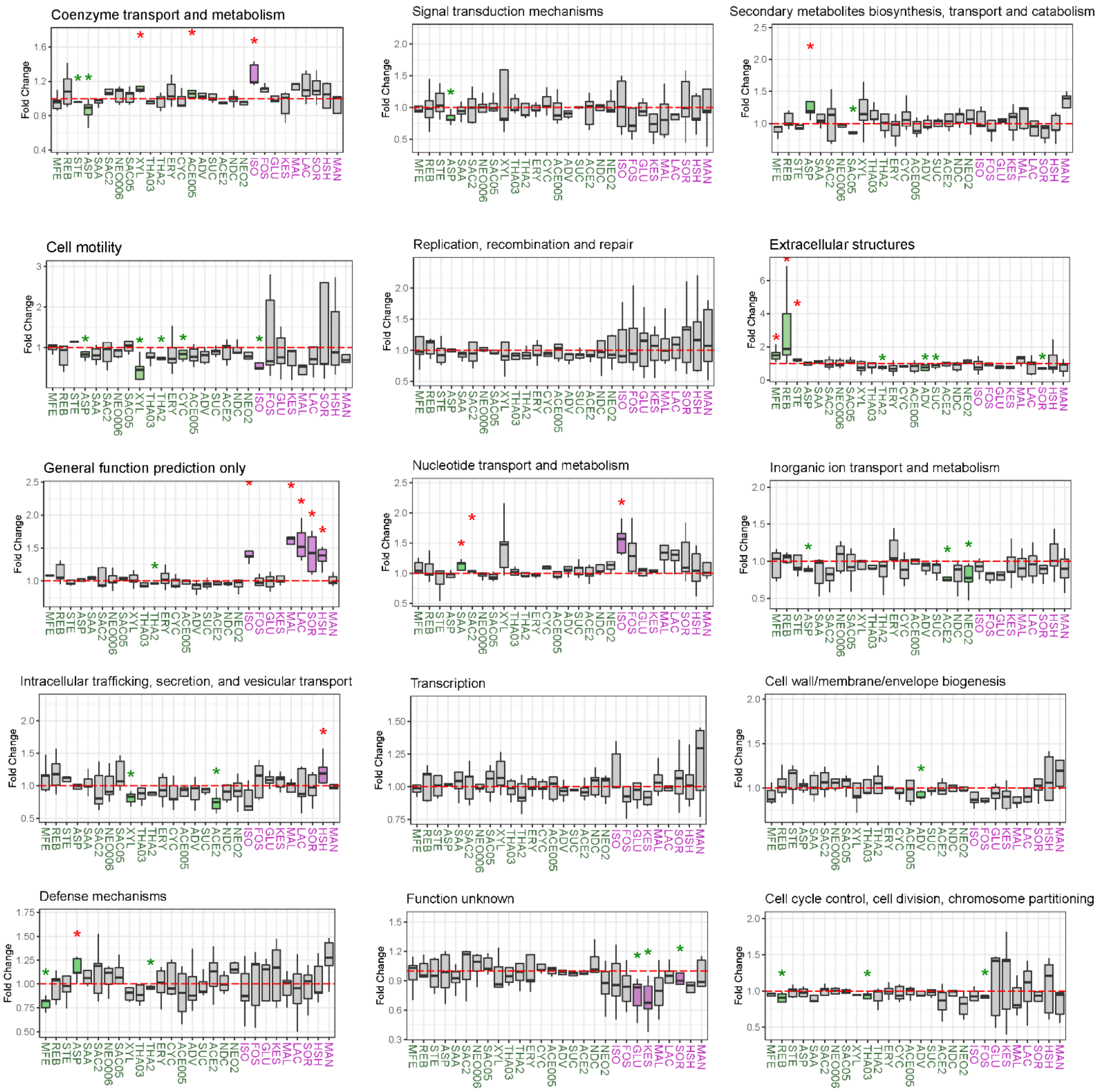

Supplementary Figure S3. Responses of other COG categories in addition to Figure 3.

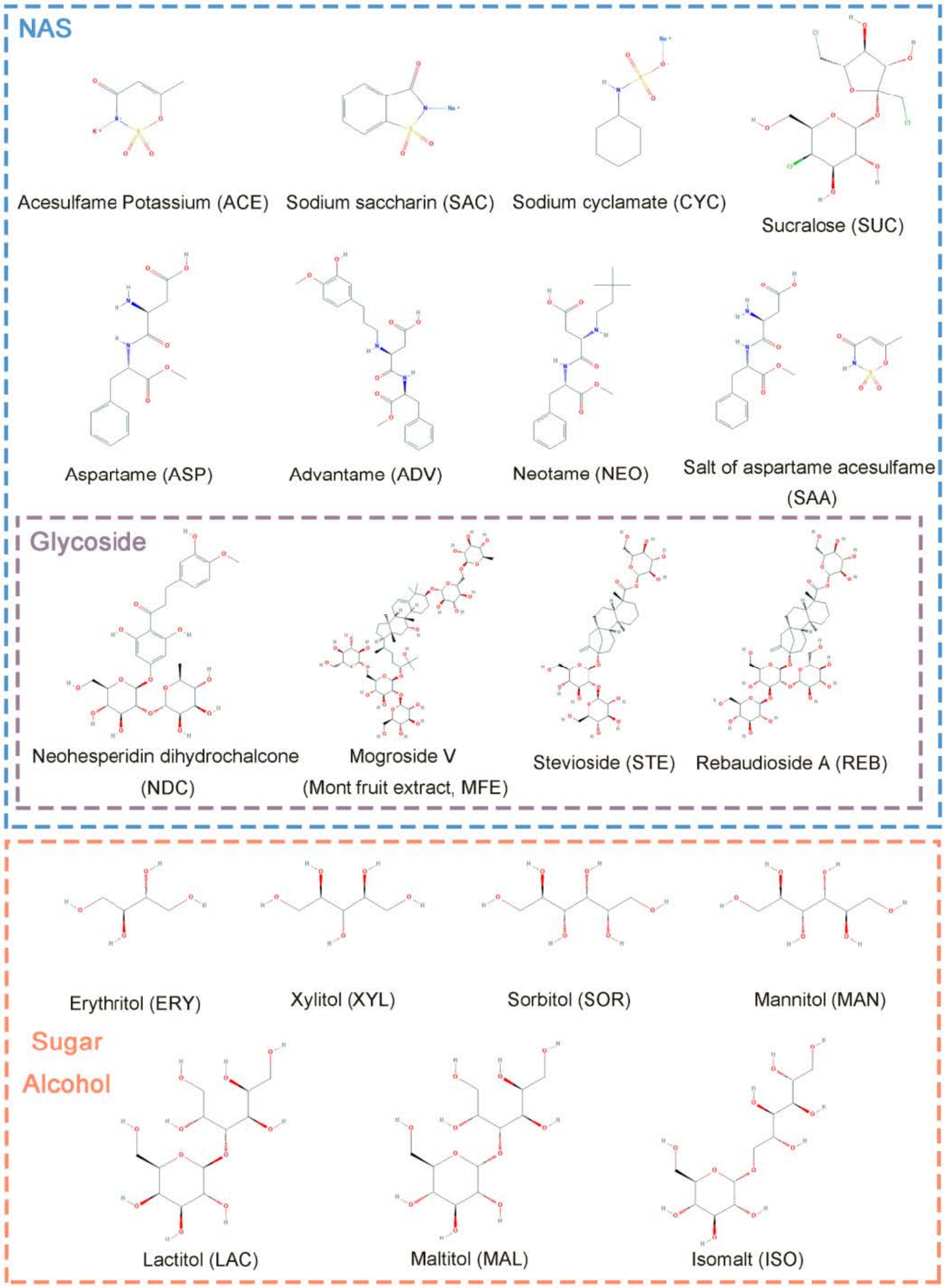

Supplementary Figure S4. Structure of sweeteners. Thaumatin (THA) and hydrogenated starch hydrolysates (HSH) were not included in this figure as they are mixtures and have no structural formula.

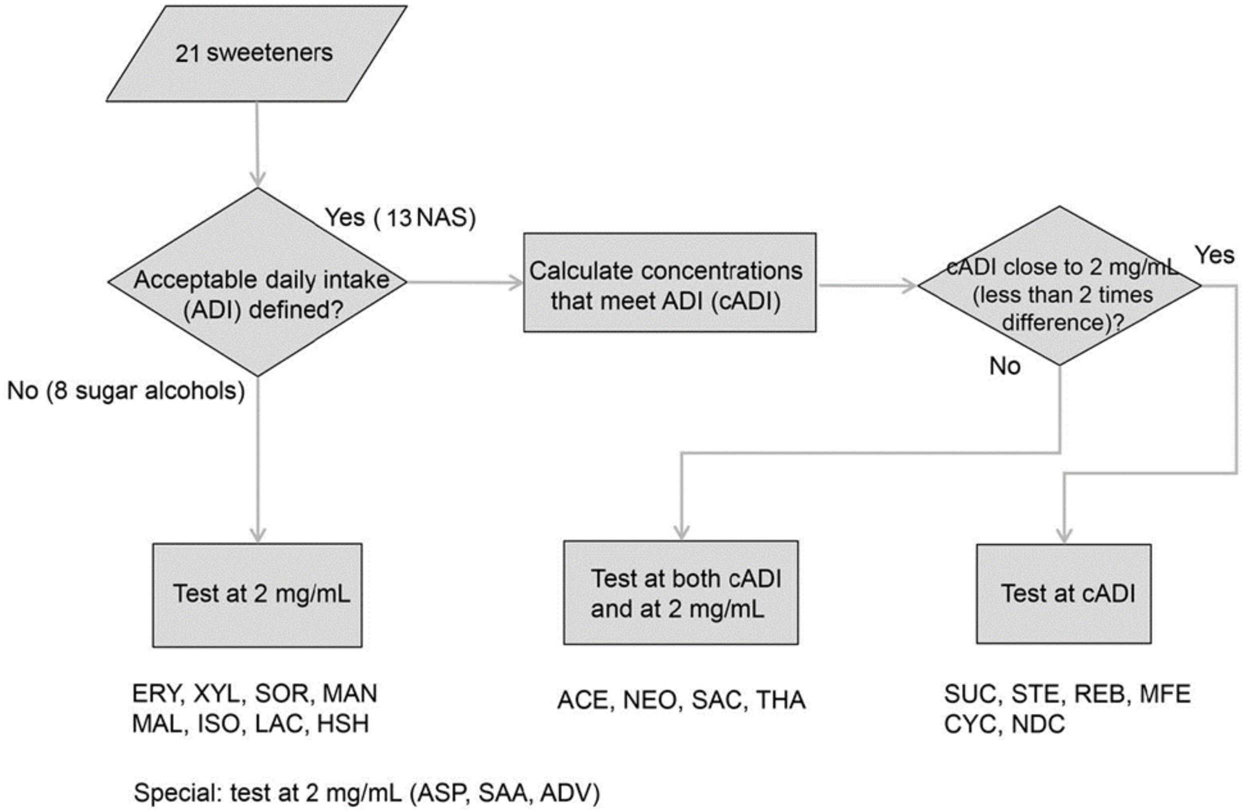

Supplementary Figure S5. Procedures used to determine sweetener concentrations.

Supplementary Table S1 Summary of sweeteners.

Sweetener*	Classification	Abbreviation in this paper	PubChem CID	Molecular Weight (g/mol)	Approved by
Acesulfame K	NAS	ACE	11074431.00	201.24	HC, EFSA, FDA
Aspartame	NAS	ASP	134601.00	294.30	HC, EFSA, FDA
Advantame	NAS	ADV	10389431.00	458.50	HC, EFSA, FDA
Neotame	NAS	NEO	9810996.00	378.50	HC, EFSA, FDA
Saccharin (Sodium saccharin dihydrate)	NAS	SAC	517320.00	241.20	HC, EFSA, FDA
Sucralose	NAS	SUC	71485.00	397.60	HC, EFSA, FDA
Stevia extract (Stevioside)	NAS	STE	442089.00	804.90	HC, EFSA, FDA
Stevia extract (Rebaudioside A)	NAS	REB	6918840.00	967.00	HC, EFSA, FDA
Monk fruit extract	NAS	MFE	N/A	N/A	HC, FDA
Thaumatin	NAS	THA	N/A	N/A	HC, EFSA, FDA
Cyclamate (Sodium cyclamate)	NAS	CYC	23665706.00	201.22	EFSA
Neohesperidin Dihydrochalcone	NAS	NDC	30231.00	612.60	EFSA
Salt of Aspartame-Acesulfame	NAS	SAA	25130065.00	457.50	EFSA
Sorbitol (D-Sorbitol)	sugar alcohols	SOR	5780.00	182.17	HC, EFSA, FDA
Mannitol (D-Mannitol)	sugar alcohols	MAN	6251.00	182.17	HC, EFSA, FDA
Isomalt	sugar alcohols	ISO	88735.00	344.31	HC, EFSA, FDA
Maltitol	sugar alcohols	MAL	493591.00	344.31	HC, EFSA, FDA
Lactitol (Lactitol monohydrate)	sugar alcohols	LAC	3067270.00	362.33	HC, EFSA, FDA
Xylitol	sugar alcohols	XYL	6912.00	152.15	HC, EFSA, FDA
Erythritol (meso-Erythritol)	sugar alcohols	ERY	222285.00	122.12	HC, EFSA, FDA
Hydrogenated starch hydrolysates	sugar alcohols	HSH	N/A	N/A	HC, EFSA, FDA
Glucose (D-Glucose)	dietary sugar (positive control)	GLU	5793.00	180.16	N/A
1-Kestose	oligosaccharide (positive control)	KES	440080.00	504.40	N/A
Fructooligosaccharide	oligosaccharide (positive control)	FOS	N/A	N/A	N/A

Supplementary Table S1 (Continued)

Sweetener*	Supplier and catalog number	Acceptable Daily Intake (ADI) (mg/kg bw/d)	cADI without considering proportion reaching the colon (mg/mL)	Proportion that reaches the colon (%)	Concentration in medium (mg/mL)
Acesulfame K	TCI A1490	15 (FDA)	0.05	1[1]	0.05 (AE005) and 2 (ACE2)
Aspartame	Alfa Aesar J61523	50 (FDA)	17.58	0[2]	2.00
Advantame	Sigma-aldrich 80054	32.8 (FDA)	10.31	89.5[3]	2.00
Neotame	Sigma-aldrich 49777	0.3 (FDA)	0.07	63.7[4]	0.067 (NEO006) and 2 (NEO2)
Saccharin (Sodium saccharin dihydrate)	J&K 926097	15 (FDA)	0.53	10[5]	0.5 (SAC05) and 2 (SAC2)
Sucralose	Alfa Aesar J66736	5 (FDA)	1.49	85[6]	1.50
Stevia extract (Stevioside)	TCI S0594	4 (FDA)	1.41	100[5]	1.40
Stevia extract (Rebaudioside A)	TCI R0095	4 (FDA)	1.41	100[5]	1.40
Monk fruit extract	Sigma USP 1445492	6.8 (FDA)**	2.39	100[5]	2.40
Thaumatin	TCI T1144	1.1 (FDA)***	0.39	0[7]	0.39 (THA03) and 2 (THA2)
Cyclamate (Sodium cyclamate)	Alfa Aesar A18666	7 (EFSA)	1.55	63[8]	1.60
Neohesperidin Dihydrochalcone	TCI N0675	5 (EFSA)	1.76	100[9]	1.80
Salt of Aspartame-Acesulfame	Sigma USP 1043750	20.46 (EFSA)****	7.19	0[8]	2.00
Sorbitol (D-Sorbitol)	TCI S0065	N/D	N/A	75[10]	2.00
Mannitol (D-Mannitol)	TCI M0044	N/D	N/A	75[10]	2.00
Isomalt	Sigma-aldrich PHR1769	N/D	N/A	90[10]	2.00
Maltitol	TCI M0797	N/D	N/A	60[10]	2.00
Lactitol (Lactitol monohydrate)	J&K 126721	N/D	N/A	98[10]	2.00
Xylitol	TCI X0018	N/D	N/A	50[10]	2.00
Erythritol (meso-Erythritol)	TCI E0021	N/D	N/A	10[10]	2.00
Hydrogenated starch hydrolysates	CarboMer Inc. 68425-17-2	N/D	N/A	60[10]	2.00
Glucose (D-Glucose)		N/A	N/A	N/A	2.00
1-Kestose		N/A	N/A	N/A	2.00
Fructooligosaccharide		N/A	N/A	N/A	2.00

*Names of the compounds that were used to represent the sweetener are shown in the brackets.

**The estimated 90th percentile intake of MFE in the general population by the FDA is used here, as its ADI is not specified[11].

*** The highest estimated exposure level of THA in the general population by the EFSA is used here, as its ADI is not specified[12].

****The ADI of SAA is calculated based on ADI of both ASP and acesulfame, as specified by the EFSA[13].

Reference:

(1) Vogt, N. M.; Kerby, R. L.; Dill-McFarland, K. A.; Harding, S. J.; Merluzzi, A. P.; Johnson, S. C.; Carlsson, C. M.; Asthana, S.; Zetterberg, H.; Blennow, K.; Bendlin, B. B.; Rey, F. E. Gut Microbiome Alterations in Alzheimer's Disease. Sci Rep 2017, 7 (1), 13537. https://doi.org/10.1038/s41598-017-13601-y.

(2) B, C.; Trugo, L.; P, F. Encyclopedia of Food Sciences and Nutrition.; 2003.

(3) EFSA Panel on Food Additives and Nutrient Sources added to Food (ANS). Scientific Opinion on the Safety of Advantame for the Proposed Uses as a Food Additive. EFS2 2013, 11 (7). https://doi.org/10.2903/j.efsa.2013.3301.

(4) Neotame as a Sweetener and Flavour Enhancer - Scientific Opinion of the Panel on Food Additives, Flavourings, Processing Aids and Materials in Contact with Food. EFSA Journal No. EFSA Journal. https://doi.org/10.2903/j.efsa.2007.581.

(5) Xu, F.; Li, D.-P.; Huang, Z.-C.; Lu, F.-L.; Wang, L.; Huang, Y.-L.; Wang, R.-F.; Liu, G.-X.; Shang, M.-Y.; Cai, S.-Q. Exploring in Vitro, in Vivo Metabolism of Mogroside V and Distribution of Its Metabolites in Rats by HPLC-ESI-IT-TOF-MSn. Journal of Pharmaceutical and Biomedical Analysis 2015, 115, 418–430. https://doi.org/10.1016/j.jpba.2015.07.024.

(6) Roberts, A.; Renwick, A. G.; Sims, J.; Snodin, D. J. Sucralose Metabolism and Pharmacokinetics in Man. Food and Chemical Toxicology 2000, 38, 31–41. https://doi.org/10.1016/S0278-6915(00)00026-0.

(7) Renwick, A. G. The Metabolism of Intense Sweeteners. Xenobiotica 1986, 16 (10–11), 1057–1071. https://doi.org/10.3109/00498258609038983.

(8) L., O.-N. Alternative Sweeteners.

(9) SCIENTIFIC OPINION Flavouring Group Evaluation 32 (FGE.32): Flavonoids (Flavanones and Dihydrochalcones) from Chemical Groups 25 and 30. EFSA Journal No. 2010; 8(9):1065. https://doi.org/10.2903/j.efsa.2010.1065.

(10) Livesey, G. Health Potential of Polyols as Sugar Replacers, with Emphasis on Low Glycaemic Properties. Nutr. Res. Rev. 2003, 16 (2), 163–191. https://doi.org/10.1079/NRR200371.

(11) GRAS Notice 627: Siraitia Grosvenorii Swingle (Luo Han Guo) Fruit Juice Concentrate; 2016.

(12) Canfora, E. E.; Jocken, J. W.; Blaak, E. E. Short-Chain Fatty Acids in Control of Body Weight and Insulin Sensitivity. Nat Rev Endocrinol 2015, 11 (10), 577–591. https://doi.org/10.1038/nrendo.2015.128.

(13) Hamer, H. M.; Jonkers, D.; Venema, K.; Vanhoutvin, S.; Troost, F. J.; Brummer, R.-J. Review Article: The Role of Butyrate on Colonic Function: REVIEW: ROLE OF BUTYRATE ON COLONIC FUNCTION. Alimentary Pharmacology & Therapeutics 2007, 27 (2), 104–119. https://doi.org/10.1111/j.1365-2036.2007.03562.x.
