# Supplementary material for: Comprehensive Assessment of Functional Effects of Commonly Used Sugar Substitute Sweeteners on Ex Vivo Human Gut Microbiome
Source: Microbiol Spectr. 2022 Jun 13;10(4):e00412-22. doi: 10.1128/spectrum.00412-22 (PMC9431030; doi:10.1128/spectrum.00412-22)
Supplement: Reviewer comments [file reviewer-comments.pdf]

AMERICAN SOCIETY FOR MICROBIOLOGY | Microbiology Spectrum

Comprehensive assessment of functional effects of commonly used sugar substitute sweeteners on *ex vivo* human gut microbiome

Zhongzhi Sun, Wenju Wang, Leyuan Li, Xu Zhang, Zhibin Ning, Janice Mayne, Krystal Walker, Alain Stintzi, and Daniel Figeys

Corresponding Author(s): Daniel Figeys, University of Ottawa

Review Timeline:

Submission Date:	February 1, 2022
Editorial Decision:	February 17, 2022
Revision Received:	March 21, 2022
Editorial Decision:	April 29, 2022
Revision Received:	May 4, 2022
Accepted:	May 21, 2022

Editor: Steven Frese

Reviewer(s): The reviewers have opted to remain anonymous.

Transaction Report:

(Note: With the exception of the correction of typographical or spelling errors that could be a source of ambiguity, letters and reports are not edited. The original formatting of letters and referee reports may not be reflected in this compilation.)

DOI: https://doi.org/10.1128/spectrum.00412-22

February 17, 2022

Dr. Daniel Figeys
University of Ottawa
Ottawa Institute of Systems Biology, Department of Biochemistry, Microbiology and Immunology
Roger Guidon Hall, Room 4207
451 Smyth Rd
Ottawa, ONT K1H 8M5
Canada

Re: Spectrum00412-22 (Comprehensive assessment of functional effects of commonly used sweeteners on ex vivo human gut microbiome)

Dear Dr. Daniel Figeys:

Thank you for submitting your manuscript to Microbiology Spectrum. When submitting the revised version of your paper, please provide (1) point-by-point responses to the issues raised by the reviewers as file type "Response to Reviewers," not in your cover letter, and (2) a PDF file that indicates the changes from the original submission (by highlighting or underlining the changes) as file type "Marked Up Manuscript - For Review Only". Please use this link to submit your revised manuscript - we strongly recommend that you submit your paper within the next 60 days or reach out to me. Detailed instructions on submitting your revised paper are below.

Link Not Available

Thank you for the privilege of reviewing your work. Below you will find instructions from the Microbiology Spectrum editorial office and comments generated during the review.

ASM policy requires that data be available to the public upon online posting of the article, so please verify all links to sequence records, if present, and make sure that each number retrieves the full record of the data. If a new accession number is not linked or a link is broken, provide production staff with the correct URL for the record. If the accession numbers for new data are not publicly accessible before the expected online posting of the article, publication of your article may be delayed; please contact the ASM production staff immediately with the expected release date.

The ASM Journals program strives for constant improvement in our submission and publication process. Please tell us how we can improve your experience by taking this quick Author Survey.

Sincerely,

Steven Frese

Editor, Microbiology Spectrum

Journals Department
American Society for Microbiology
1752 N St., NW
Washington, DC 20036
E-mail: spectrum@asmusa.org

Reviewer comments:

Reviewer #1 (Comments for the Author):

In this manuscript, the authors perform proteomic analysis of anaerobic fecal microbiome cultures (from five human donors), supplemented with a broad panel of various alternatives to caloric sugars, including non-caloric artificial sweeteners (NAS), steviol glycosides, and sugar alcohols. Under these specific experimental conditions (a caveat that is insufficiently

discussed), they report that sugar alcohols, but not NAS (for the most part), can impact the proteome of these cultures. This part of the manuscript is solid but under-explored (see below). Much of the manuscript beyond this point is based on predicting microbial composition from the proteomic data. However, the authors do not provide validation to this approach, and I find the results insufficiently convincing, speculative, and confusing.

Specific comments

1. Throughout the manuscript, the authors need to be more explicit about the specific methodology and statistical tests used (see comments below).
2. The authors should provide, through a figure or supplementary table, a list of all the proteins that were significantly altered per a given sweetener treatment. This addition will be an extremely valuable resource that would further our understanding of microbiome-sweetener interactions. I do not find the current global measurements of proteomics-based distances sufficiently informative.
3. There are insufficient details in the main text or the methods section regarding the approach/pipeline used by the authors to infer microbial composition from metaproteomics. If this is based on a validated approach, the authors need to add the relevant reference(s). If this is a new method, the authors need to provide a head-to-head comparison of metagenomics profiling to the composition inferred from metaproteomics. If neither is available, this section should be removed.
4. I find the section titled "CHO" cluster sweeteners had prebiotic-like effects on Clostridia" problematic, as the authors have not performed a compositional analysis; rather, composition is inferred from metaproteomics (see above comment). Furthermore, describing compositional impacts at the class level seems too crude, especially for such a diverse bacterial class that includes known pathogens (C. difficile, C. tetani...) and commensals associated with health benefits. Do the authors suggest that these sweeteners have "prebiotic" impacts on pathogens? The use of the term "prebiotic" throughout the manuscript may be misleading or confusing. According to the ISAPP expert consensus statement (https://www.nature.com/articles/nrgastro.2017.75), a prebiotic is "a substrate that is selectively utilized by host microorganisms conferring a health benefit". The authors do not show that the effects of these sweeteners on the microbiome confer any health benefit and, in fact, mention works that implicate their potential negative impacts on health. The term "prebiotic" is therefore not suitable throughout the manuscript; a more nuanced term (e.g., "microbiome-modulating additives") would be more appropriate. If the authors want to discuss the effects of sweeteners on composition, they should choose a more suitable method. The overall message of this section regarding sugar alcohols is confusing. The authors mention that these sweeteners may be good options as prebiotics for Clostridia growth. But they also confirm that these sweeteners alter the functional profile of the gut in a way that causes increased susceptibility to abdominal pain, bloating, gas, etc.
5. The authors should include PBS controls/triplicates on all figures involving clustering? It would be essential to understand where the controls cluster in these figures, to understand which sweeteners are most similar to / different from controls. In addition, in Figure 1B, can the authors show the boxplots for PBS triplicates (The comparison being performed for statistical significance).
6. The authors note that "While we did not observe any global effects of saccharin on the metaproteome using our in vitro assay, we observed changes at the individual level indicating an individual-specific response to saccharin." - Can they elaborate on the difference between the two here and point directly to its support in the data? For example, in Figure 1A, they do report statistically significant differences in specific genera between saccharin samples and control, even if the Bray-Curtis differences are not statistically significant compared to controls. In Figure S1, it looks like for the sweeteners in the "NAS" cluster, there is a significant increase in variation between replicates compared to the PBS controls - does this mean that there were significant effects, but these effects varied greatly depending on the starting microbiome?
7. For a study testing multiple different sweeteners, the authors present a reasonable approach for determining which concentrations to use. Importantly, I could not find in the manuscript any discussion of null effects being a result of the chosen dose. Indeed, for sweeteners in which more than one dose was assessed (e.g., saccharin), the authors see dose-dependent impacts, which suggests that other doses would have a stronger impact. Since the authors report a null phenotype for some of the sweeteners, discussing this caveat is important.
8. This section of the discussion is somewhat misleading "the comparison of the effects of a large number of different sweeteners on human gut microbes has not been reported. In this study, we investigated the taxonomic and functional responses of five individual human microbiomes" as the authors did not study the effect of the sweeteners in humans, but rather in culture. It is unclear whether similar impacts will be observed (or not observed) in the gut itself. It is also unclear how many of the gut bacteria in the original sample survive the passage to the anaerobic environment, and how many of these are able to grow in these experimental conditions. There is value in studying these in vitro interactions, but the authors need to be more cautious in their interpretation and include these caveats in their discussion.
9. I'm wondering whether a more refined terminology can be used instead of generic "sweeteners", which would also apply to caloric sweeteners such as sucrose, glucose, fructose? For example, the use of the term sweeteners is confusing in line 52: "Sweeteners are food additives used to increase the sweetness of food while contributing a negligible amount of energy". Are all sweeteners in this study high-intensity sweeteners? Are they all low-calorie sweeteners (LCS)? The authors use "artificial sweeteners" in their keywords but some of the tested substrates are not artificial. Somewhat relevant, The authors use the definition "glycoside-type NAS" (e.g., in the legend for figure 1), but steviol glycosides are not NAS (i.e., they are not artificial).
10. The authors emphasize the distinction between the length of the carbon backbone in two noted clusters, but is there any evidence that the discrepancy in response stems from the length of the carbon backbone? If not, I do think that the authors should note this distinction but perhaps it's better if this is toned-down.

11. The parts of the introduction that describe a potential link between sweeteners and metabolic syndrome need to be more balanced. E.g., the authors write that "many studies have linked their consumption with the development of diseases and metabolic syndrome", but the references provided are for associative studies that cannot determine a causal link. References to RCTs would be more appropriate here, however the authors should mention that there are also RCTs that did not find a negative effect, or even reported a benefit. Similarly, the authors state that "Sweeteners have been found to induce changes in the gut microbiome composition in animals and humans by metagenomics", but there are a few studies in humans that did not find an effect on the microbiome (although using 16S, e.g., Serrano 2021). Balancing the introduction does not discount the observations made by the authors. In fact, it would be interesting to explore potential personalized effects, which have been suggested to mediate opposing outcomes between RCTs with sweeteners.
12. This sentence in the introduction "To date, there have been no systematic studies on sweeteners for their effects on the microbiome" seems a little vague and unnecessarily minimizing previous work - is there a better way to specifically highlight the uniqueness of this report?
13. I'm curious how the authors defined "non-sweetener consumer". The panel included quite a few sweeteners that can be found in a wide range of products. E.g., xylitol is found in many products, like gum, toothpaste, peanut butter / other condiments, at varying levels. Some of these sweeteners also occur naturally in some foods. Was this done in any systematic manner? FFQs? When the volunteers were given a self-assessment on sweetener intake, were they made aware of how prevalent these sweeteners are before determining their own sweetener intake? Were they supplied with a (very extensive) list of items containing these sweeteners?

Additional comments
Consider using the full names for sweeteners instead of acronyms throughout the text, it would make it much more accessible (as well as make the study identifiable in keyword searches for each sweetener).

Line 17: Consider changing to "Gut microbiome composition and function are often associated with health and disease status."
Line 26: Consider rewording this sentence so it is clear exactly what the two clusters are clustering.
Line 30: What do the authors mean by "similar" here? Consider being more specific in the wording so it is clear to the reader exactly what analysis and comparison is being performed.
Line 37: Consider changing to "The Human gut microbiome"
Line 68: Missing period after "microbiome".
Line 109: Briefly define DC assay here in the text
Line 134: What is "CHO"? Define at first use in the text
Line 158 - what are "In addition, proteins with only general function prediction"?
Line 183 - Use complete names for COG categories
Line 251- "clustered sweeteners might promote the cell division of Clostridia." - is the more direct implication of this increased cellular growth?
Line 311: "deep-well plates containing pre-reduced, optimized microbiome culture medium" Can the authors describe the type of media used directly here in the text instead of in the reference?
Line 363-365: Can the authors provide more details on the type of protein search/alignment performed? Were there e-value, percent identity, or mismatch cutoffs required to identify a protein fragment hit? How are protein fragments that map equally well to two different proteins handled? Two different taxa?
Line 370- "DESq2" typo.
Line 381 - what database was used for taxonomic assignment, and what taxonomic framework? (NCBI, etc.)
Figure 2- "Significantly affected genus" should be "genera" - How is significance determined here? Is it the same as Wilcoxon rank-sum in 2B?

Reviewer #2 (Public repository details (Required)):

I confirmed that a dataset has been deposited at ProteomeXchange under the accession number provided. However, access is not public, so I did not confirm the nature of the dataset.

Reviewer #2 (Comments for the Author):

General comments:
1. It is a little confusing to use two different ways of "counting" the data prior to clustering (genus biomass, and predicted protein function), especially since they gave such different results. I understand why the authors conducted the analyses this way (it gives a microbiota structure and then function flow to the paper), but it does leave the reader wanting to understand how to

relate the two analyses. Additionally, the explanation in the Discussion section lines 224 - 228 leaves the reader wondering why the analysis of the microbiota structure using genus biomass was performed at all. At a minimum the genera that are members of class Clostridia could be indicated in lines 122 - 128. Similarly, the genera within class Clostridia that showed differential expression of predicted protein functions could be mentioned in line 181. Maximally, the genus biomass section could be omitted.
2. The discussion section is predominantly a restatement of the results. It would be interesting to read more about how these results fit into the existing body of literature on carbohydrate interactions with the gut microbiota.
3. There doesn't seem to be any information on the expression of carbohydrate active enzymes. Is it not possible to get that data from metaproteomes?

Specific Comments:
1. Line 134: It seems like CHO is referring to the carbohydrates in that cluster, but I don't see the acronym defined anywhere.
2. Lines 183 - 184: Please describe the COG categories, rather than stating the letter designation.
3. Line 303: "accessed" should be "assessed"
4. Line 311: The complete final recipe for the microbiome culture media doesn't seem to be in either of the cited references. It would be very helpful to the reader to add a supplementary table (or some additional text in the methods section) describing the details of the recipe.

Staff Comments:

Preparing Revision Guidelines
To submit your modified manuscript, log onto the eJP submission site at https://spectrum.msubmit.net/cgi-bin/main.plex. Go to Author Tasks and click the appropriate manuscript title to begin the revision process. The information that you entered when you first submitted the paper will be displayed. Please update the information as necessary. Here are a few examples of required updates that authors must address:

• Point-by-point responses to the issues raised by the reviewers in a file named "Response to Reviewers," NOT IN YOUR COVER LETTER.
• Upload a compare copy of the manuscript (without figures) as a "Marked-Up Manuscript" file.
• Each figure must be uploaded as a separate file, and any multipanel figures must be assembled into one file.
• Manuscript: A .DOC version of the revised manuscript
• Figures: Editable, high-resolution, individual figure files are required at revision, TIFF or EPS files are preferred

For complete guidelines on revision requirements, please see the journal Submission and Review Process requirements at https://journals.asm.org/journal/Spectrum/submission-review-process. **Submissions of a paper that does not conform to Microbiology Spectrum guidelines will delay acceptance of your manuscript.** "

Please return the manuscript within 60 days; if you cannot complete the modification within this time period, please contact me. If you do not wish to modify the manuscript and prefer to submit it to another journal, please notify me of your decision immediately so that the manuscript may be formally withdrawn from consideration by Microbiology Spectrum.

If your manuscript is accepted for publication, you will be contacted separately about payment when the proofs are issued; please follow the instructions in that e-mail. Arrangements for payment must be made before your article is published. For a complete list of **Publication Fees**, including supplemental material costs, please visit ourwebsite.

Corresponding authors may join or renew ASM membership to obtain discounts on publication fees. Need to upgrade your membership level? Please contact Customer Service at Service@asmusa.org.

Thank you for submitting your paper to Microbiology Spectrum.

In this manuscript, the authors perform proteomic analysis of anaerobic fecal microbiome cultures (from five human donors), supplemented with a broad panel of various alternatives to caloric sugars, including non-caloric artificial sweeteners (NAS), steviol glycosides, and sugar alcohols. Under these specific experimental conditions (a caveat that is insufficiently discussed), they report that sugar alcohols, but not NAS (for the most part), can impact the proteome of these cultures. This part of the manuscript is solid but under-explored (see below). Much of the manuscript beyond this point is based on predicting microbial composition from the proteomic data. However, the authors do not provide validation to this approach, and I find the results insufficiently convincing, speculative, and confusing.

Specific comments

1. Throughout the manuscript, the authors need to be more explicit about the specific methodology and statistical tests used (see comments below).
2. The authors should provide, through a figure or supplementary table, a list of **all** the proteins that were significantly altered per a given sweetener treatment. This addition will be an extremely valuable resource that would further our understanding of microbiome-sweetener interactions. I do not find the current global measurements of proteomics-based distances sufficiently informative.
3. There are insufficient details in the main text or the methods section regarding the approach/pipeline used by the authors to infer microbial composition from metaproteomics. If this is based on a validated approach, the authors need to add the relevant reference(s). If this is a new method, the authors need to provide a head-to-head comparison of metagenomics profiling to the composition inferred from metaproteomics. If neither is available, this section should be removed.
4. I find the section titled "CHO" cluster sweeteners had prebiotic-like effects on Clostridia" problematic, as the authors have not performed a compositional analysis; rather, composition is inferred from metaproteomics (see above comment). Furthermore, describing compositional impacts at the class level seems too crude, especially for such a diverse bacterial class that includes known pathogens (*C. difficile, C. tetani*…) and commensals associated with health benefits. Do the authors suggest that these sweeteners have "prebiotic" impacts on pathogens? The use of the term "prebiotic" throughout the manuscript may be misleading or confusing. According to the ISAPP expert consensus statement (https://www.nature.com/articles/nrgastro.2017.75), a prebiotic is "a substrate that is selectively utilized by host microorganisms conferring a health benefit". The authors do not show that the effects of these sweeteners on the microbiome confer any health benefit and, in fact, mention works that implicate their potential negative impacts on health. The term "prebiotic" is therefore not suitable throughout the manuscript; a more nuanced term (e.g., "microbiome-modulating additives") would be more appropriate. If the authors want to discuss the effects of sweeteners on composition, they should choose a more suitable method. The overall message of this section regarding sugar alcohols is confusing. The authors mention that

these sweeteners may be good options as prebiotics for Clostridia growth. But they also confirm that these sweeteners alter the functional profile of the gut in a way that causes increased susceptibility to abdominal pain, bloating, gas, etc.

5. The authors should include PBS controls/triplicates on all figures involving clustering? It would be essential to understand where the controls cluster in these figures, to understand which sweeteners are most similar to / different from controls. In addition, in Figure 1B, can the authors show the boxplots for PBS triplicates (The comparison being performed for statistical significance).
6. The authors note that "While we did not observe any global effects of saccharin on the metaproteome using our in vitro assay, we observed changes at the individual level indicating an individual-specific response to saccharin." - Can they elaborate on the difference between the two here and point directly to its support in the data? For example, in Figure 1A, they do report statistically significant differences in specific genera between saccharin samples and control, even if the Bray-Curtis differences are not statistically significant compared to controls. In Figure S1, it looks like for the sweeteners in the "NAS" cluster, there is a significant increase in variation between replicates compared to the PBS controls - does this mean that there were significant effects, but these effects varied greatly depending on the starting microbiome?
7. For a study testing multiple different sweeteners, the authors present a reasonable approach for determining which concentrations to use. Importantly, I could not find in the manuscript any discussion of null effects being a result of the chosen dose. Indeed, for sweeteners in which more than one dose was assessed (e.g., saccharin), the authors see dose-dependent impacts, which suggests that other doses would have a stronger impact. Since the authors report a null phenotype for some of the sweeteners, discussing this caveat is important.
8. This section of the discussion is somewhat misleading "the comparison of the effects of a large number of different sweeteners on human gut microbes has not been reported. In this study, we investigated the taxonomic and functional responses of five individual human microbiomes" as the authors did not study the effect of the sweeteners in humans, but rather in culture. It is unclear whether similar impacts will be observed (or not observed) in the gut itself. It is also unclear how many of the gut bacteria in the original sample survive the passage to the anaerobic environment, and how many of these are able to grow in these experimental conditions. There is value in studying these *in vitro* interactions, but the authors need to be more cautious in their interpretation and include these caveats in their discussion.
9. I'm wondering whether a more refined terminology can be used instead of generic "sweeteners", which would also apply to caloric sweeteners such as sucrose, glucose, fructose? For example, the use of the term sweeteners is confusing in line 52: "Sweeteners are food additives used to increase the sweetness of food while contributing a negligible amount of energy". Are all sweeteners in this study high-intensity sweeteners? Are they all low-calorie sweeteners (LCS)? The authors use "artificial

sweeteners" in their keywords but some of the tested substrates are not artificial. Somewhat relevant, The authors use the definition "glycoside-type NAS" (e.g., in the legend for figure 1), but steviol glycosides are not NAS (i.e., they are not artificial).

10. The authors emphasize the distinction between the length of the carbon backbone in two noted clusters, but is there any evidence that the discrepancy in response stems from the length of the carbon backbone? If not, I do think that the authors should note this distinction but perhaps it's better if this is toned-down.
11. The parts of the introduction that describe a potential link between sweeteners and metabolic syndrome need to be more balanced. E.g., the authors write that "many studies have linked their consumption with the development of diseases and metabolic syndrome", but the references provided are for associative studies that cannot determine a causal link. References to RCTs would be more appropriate here, however the authors should mention that there are also RCTs that did not find a negative effect, or even reported a benefit. Similarly, the authors state that "Sweeteners have been found to induce changes in the gut microbiome composition in animals and humans by metagenomics", but there are a few studies in humans that did not find an effect on the microbiome (although using 16S, e.g., Serrano 2021). Balancing the introduction does not discount the observations made by the authors. In fact, it would be interesting to explore potential personalized effects, which have been suggested to mediate opposing outcomes between RCTs with sweeteners.
12. This sentence in the introduction "To date, there have been no systematic studies on sweeteners for their effects on the microbiome" seems a little vague and unnecessarily minimizing previous work – is there a better way to specifically highlight the uniqueness of this report?
13. I'm curious how the authors defined "non-sweetener consumer". The panel included quite a few sweeteners that can be found in a wide range of products. E.g., xylitol is found in many products, like gum, toothpaste, peanut butter / other condiments, at varying levels. Some of these sweeteners also occur naturally in some foods. Was this done in any systematic manner? FFQs? When the volunteers were given a self-assessment on sweetener intake, were they made aware of how prevalent these sweeteners are before determining their own sweetener intake? Were they supplied with a (very extensive) list of items containing these sweeteners?

Additional comments
Consider using the full names for sweeteners instead of acronyms throughout the text, it would make it much more accessible (as well as make the study identifiable in keyword searches for each sweetener).

Line 17: Consider changing to "Gut microbiome composition and function are often associated with health and disease status."
Line 26: Consider rewording this sentence so it is clear exactly what the two clusters are clustering.

Line 30: What do the authors mean by "similar" here? Consider being more specific in the wording so it is clear to the reader exactly what analysis and comparison is being performed.
Line 37: Consider changing to "The Human gut microbiome"
Line 68: Missing period after "microbiome".
Line 109: Briefly define DC assay here in the text
Line 134: What is "CHO"? Define at first use in the text
Line 158 - what are "In addition, proteins with only general function prediction"?
Line 183 - Use complete names for COG categories
Line 251- "clustered sweeteners might promote the cell division of Clostridia." - is the more direct implication of this increased cellular growth?
Line 311: "deep-well plates containing pre-reduced, optimized microbiome culture medium" Can the authors describe the type of media used directly here in the text instead of in the reference?
Line 363-365: Can the authors provide more details on the type of protein search/alignment performed? Were there e-value, percent identity, or mismatch cutoffs required to identify a protein fragment hit? How are protein fragments that map equally well to two different proteins handled? Two different taxa?
Line 370- "DESq2" typo.
Line 381 - what database was used for taxonomic assignment, and what taxonomic framework? (NCBI, etc.)
Figure 2- "Significantly affected genus" should be "genera" - How is significance determined here? Is it the same as Wilcoxon rank-sum in 2B?

Reviewer comments:

Reviewer #1 (Comments for the Author):

In this manuscript, the authors perform proteomic analysis of anaerobic fecal microbiome cultures (from five human donors), supplemented with a broad panel of various alternatives to caloric sugars, including non-caloric artificial sweeteners (NAS), steviol glycosides, and sugar alcohols. Under these specific experimental conditions (a caveat that is insufficiently discussed), they report that sugar alcohols, but not NAS (for the most part), can impact the proteome of these cultures. This part of the manuscript is solid but under-explored (see below). Much of the manuscript beyond this point is based on predicting microbial composition from the proteomic data. However, the authors do not provide validation to this approach, and I find the results insufficiently convincing, speculative, and confusing.

Response: Thank you for reviewing our manuscript! We have received many helpful comments for the improvement of our manuscript. We carefully revised our manuscript, especially in the result section. We hope you find the revised version satisfactory.

Specific comments

1. Throughout the manuscript, the authors need to be more explicit about the specific methodology and statistical tests used (see comments below).

Response: We gave more details about methodology in the revised manuscript. Please see the following responses and the revised manuscript.

2. The authors should provide, through a figure or supplementary table, a list of all the proteins that were significantly altered per a given sweetener treatment. This addition will be an extremely valuable resource that would further our understanding of microbiome-sweetener interactions. I do not find the current global measurements of proteomics-based distances sufficiently informative.

Response: We added a figure (Figure 1F) to show the number of significantly altered proteins under different treatments, as well as a supplementary table (Supplementary Table S3) to show the list of significantly altered proteins.

3. There are insufficient details in the main text or the methods section regarding the approach/pipeline used by the authors to infer microbial composition from metaproteomics. If this is based on a validated approach, the authors need to add the relevant reference(s). If this is a new method, the authors need to provide a head-to-head comparison of metagenomics profiling to the composition inferred from metaproteomics. If neither is available, this section should be removed.

Response: Our previous usage of the word *microbial composition* might cause misunderstanding, and we have changed the word to ***genus-level protein biomass composition*** in the revised manuscript. The species biomass contributions in microbial communities via metaproteomics has been previously assessed (Kleiner *et al.*, Nature Communications, 2017). This study has shown that although the sequencing-based

methods and metaproteomics can provide very different pictures of a community, metaproteomics-based biomass assessment is a powerful approach that allows accurate quantification of the proteinaceous biomass of a large number of taxa and provides an additional, independent measure of community structure. The genus-level protein biomass composition was acquired based on the taxonomy information of the identified peptides from MetaLab (version 1.2.0), and we pointed that in the *Microbial taxonomic analysis* section in the revised manuscript.

4. I find the section titled "CHO" cluster sweeteners had prebiotic-like effects on Clostridia" problematic, as the authors have not performed a compositional analysis; rather, composition is inferred from metaproteomics (see above comment). Furthermore, describing compositional impacts at the class level seems too crude, especially for such a diverse bacterial class that includes known pathogens (C. difficile, C. tetani...) and commensals associated with health benefits. Do the authors suggest that these sweeteners have "prebiotic" impacts on pathogens? The use of the term "prebiotic" throughout the manuscript may be misleading or confusing. According to the ISAPP expert consensus statement (https://www.nature.com/articles/nrgastro.2017.75), a prebiotic is "a substrate that is selectively utilized by host microorganisms conferring a health benefit". The authors do not show that the effects of these sweeteners on the microbiome confer any health benefit and, in fact, mention works that implicate their potential negative impacts on health. The term "prebiotic" is therefore not suitable throughout the manuscript; a more nuanced term (e.g., "microbiome-modulating additives") would be more appropriate. If the authors want to discuss the effects of sweeteners on composition, they should choose a more suitable method. The overall message of this section regarding sugar alcohols is confusing. The authors mention that these sweeteners may be good options as prebiotics for Clostridia growth. But they also confirm that these sweeteners alter the functional profile of the gut in a way that causes increased susceptibility to abdominal pain, bloating, gas, etc.

Response: "Microbiome-modulating additives" is a good suggestion. We first used the word "prebiotic" because the functional responses of "CHO" cluster sweeteners were well clustered with well-known prebiotics FOS and KES. However, more evidence might be needed to prove the prebiotic effects of the "CHO" cluster. For this consideration, we changed the title of this part into '***"CHO" cluster sweeteners modulated the metabolism of Clostridia***'. And we reduced the mention of prebiotics in the revised manuscript.

Our result revealed that the sweetener-induced metaproteomic responses of individual microbiomes had two major patterns. Of the proteins that best discriminate two patterns, most were from Clostridia. These proteins can be divided into two groups, *CHO elevated group* which had higher abundance in the "CHO" cluster and *CHO depleted group* which had higher abundance in the "NAS" cluster. Proteins from the two groups had different functions, showing the modulating pattern of the "CHO" cluster sweeteners on the Clostridia. Although we did not reveal a more specific pattern on the lower taxonomic level, the trends in overall changes in Clostridia might still be informative. The enrichment of iron-dependent pathways in TCA cycle and oxidative phosphorylation indicated increased cellular energy metabolism in Clostridia in response to the "CHO" cluster. The

list of proteins from both CHO elevated group and CHO depleted group could be a good reference for further studies on the response of sweeteners of Clostridia.

5. The authors should include PBS controls/triplicates on all figures involving clustering? It would be essential to understand where the controls cluster in these figures, to understand which sweeteners are most similar to / different from controls. In addition, in Figure 1B, can the authors show the boxplots for PBS triplicates (The comparison being performed for statistical significance).

Response: In Figure 1B, we added the calculated average Bray-Curtis distance between PBS triplicates. The distances from 5 individuals can be used as a useful comparison. However, as clustering results in Figure 2 and Figure 3 were calculated based on the fold change of genus-level protein abundance and averaged $\log_2$ fold change of COG abundances between the sweetener-treated group and PBS group, including PBS controls in these figures might not be informative. In Figure 4B, the PBS was included in the cluster as the cluster was based on the intensity of discriminative proteins. To understand which sweeteners are most similar to / different from controls, the added figure (Figure 1F) and supplementary table (Table S3) for significantly altered proteins and the previous PCA plots would be a good resource.

6. The authors note that "While we did not observe any global effects of saccharin on the metaproteome using our in vitro assay, we observed changes at the individual level indicating an individual-specific response to saccharin." - Can they elaborate on the difference between the two here and point directly to its support in the data? For example, in Figure 1A, they do report statistically significant differences in specific genera between saccharin samples and control, even if the Bray-Curtis differences are not statistically significant compared to controls. In Figure S1, it looks like for the sweeteners in the "NAS" cluster, there is a significant increase in variation between replicates compared to the PBS controls - does this mean that there were significant effects, but these effects varied greatly depending on the starting microbiome?

Response: We pointed to the support data in the revised version. It is indeed that for the sweeteners in the "NAS" cluster, it looks like there is a significant increase in variation between replicates compared to the PBS controls. However, the PBS controls are still well-clustered. As a result, rather than these effects varied greatly depending on the starting microbiome, we believe that it was caused by individual differences. Add we added "**From the PCA plots, we observed that compared with PBS controls, there were larger variation between replicates under the treatment of "NAS" cluster sweeteners. This suggests inter-individual differences in the response of the "NAS" cluster sweeteners."** in the discussion section.

7. For a study testing multiple different sweeteners, the authors present a reasonable approach for determining which concentrations to use. Importantly, I could not find in the manuscript any discussion of null effects being a result of the chosen dose. Indeed, for sweeteners in which more than one dose was assessed (e.g., saccharin), the authors see dose-dependent impacts, which suggests that other doses would have a stronger impact. Since the authors report a null phenotype for some of the sweeteners, discussing this caveat is

important.

Response: Thank you for your reminder. We added the following sentence in the discussion part: "**It is noted that only one or two concentrations of each sweetener were tested in this study. The number of significantly altered protein groups in SAC2 was larger than that in SAC05. And the same situation happened between THA2 and THA03, NEO2 and NEO006, as well as ACE2 and ACE005 (Figure 2), which indicated that a higher concentration of sweeteners could likely have a larger impact on the human gut microbiome. The impacts of different concentrations of sweeteners need further investigation**".

8. This section of the discussion is somewhat misleading "the comparison of the effects of a large number of different sweeteners on human gut microbes has not been reported. In this study, we investigated the taxonomic and functional responses of five individual human microbiomes" as the authors did not study the effect of the sweeteners in humans, but rather in culture. It is unclear whether similar impacts will be observed (or not observed) in the gut itself. It is also unclear how many of the gut bacteria in the original sample survive the passage to the anaerobic environment, and how many of these are able to grow in these experimental conditions. There is value in studying these in vitro interactions, but the authors need to be more cautious in their interpretation and include these caveats in their discussion.

Response: We changed the sentence into "the comparison of the effects of a large number of different sugar substitute sweeteners on human gut microbes has not been reported. In this study, we investigated the taxonomic and functional responses of five individuals' ***in-vitro cultured*** human gut microbiomes to 21 common sugar substitute sweeteners". And we have added "**We used a previously developed in vitro model which was shown to maintain microbial taxon and function. We tried to use this in vitro model to reveal the real functional change in the human gut microbiome. This in vitro model has been shown to recapitulate microbiome changes observed in vivo. The purpose of the in vitro model is to better understand the direct effects of compounds on individual microbiomes and as with any assays would need in vivo confirmation.**" in the discussion part. We hope these will be able to tell both the benefits and limitations of the *in vitro* model.

9. I'm wondering whether a more refined terminology can be used instead of generic "sweeteners", which would also apply to caloric sweeteners such as sucrose, glucose, fructose? For example, the use of the term sweeteners is confusing in line 52: "Sweeteners are food additives used to increase the sweetness of food while contributing a negligible amount of energy". Are all sweeteners in this study high-intensity sweeteners? Are they all low-calorie sweeteners (LCS)? The authors use "artificial sweeteners" in their keywords but some of the tested substrates are not artificial. Somewhat relevant, The authors use the definition "glycoside-type NAS" (e.g., in the legend for figure 1), but steviol glycosides are not NAS (i.e., they are not artificial).

Response: We have changed the terms 'sweeteners' and 'artificial sweeteners' into '***sugar substitute sweeteners***' to differentiate sweeteners such as glucose, sucrose and fructose with sweeteners that are compared in this study. Most of the sweeteners in this

study are low-calorie sweeteners. And we have changed the sentence into: "**Sugar substitute sweeteners are food additives used to increase the sweetness of food while contributing a modest amount of energy compared to sugar.**".

10. The authors emphasize the distinction between the length of the carbon backbone in two noted clusters, but is there any evidence that the discrepancy in response stems from the length of the carbon backbone? If not, I do think that the authors should note this distinction but perhaps it's better if this is toned-down.

Response: We made some modifications in the two places where we mentioned the "backbone". In the abstract part, we changed our previous sentence to "**The first "NAS" cluster was composed of non-caloric artificial sweeteners (NAS) and two sugar alcohols with shorter carbon backbones (4-5 carbon atoms), and the second "CHO" (Carbohydrate) cluster was composed of the remaining sugar alcohols**". In the result part, we changed the sentence to "**In the "NAS" cluster, all NASs were included, plus sugar alcohols erythritol and xylitol which have shorter carbon backbones (Supplementary Figure S4)**". We hope these modifications can help to note the distinction between the length of the carbon backbone without overemphasizing it.

11. The parts of the introduction that describe a potential link between sweeteners and metabolic syndrome need to be more balanced. E.g., the authors write that "many studies have linked their consumption with the development of diseases and metabolic syndrome", but the references provided are for associative studies that cannot determine a causal link. References to RCTs would be more appropriate here, however the authors should mention that there are also RCTs that did not find a negative effect, or even reported a benefit. Similarly, the authors state that "Sweeteners have been found to induce changes in the gut microbiome composition in animals and humans by metagenomics", but there are a few studies in humans that did not find an effect on the microbiome (although using 16S, e.g., Serrano 2021). Balancing the introduction does not discount the observations made by the authors. In fact, it would be interesting to explore potential personalized effects, which have been suggested to mediate opposing outcomes between RCTs with sweeteners.

Response: To be more cautious, we changed the sentence into "Despite the proposed health benefits of sweeteners, many studies have found **associations between** their consumption and the development of diseases and metabolic syndrome, some of which were initially intended to be prevented by the use of sweeteners". And we also added the discussion about the study that found no effect of sweetener on the microbiome. "**However, Serrano *et al.* found that gut microbial diversity and composition at any taxonomic level, as well as glucose intolerance, were not altered by the high-dose saccharin supplementation in healthy humans and mice using 16S rRNA sequencing**".

12. This sentence in the introduction "To date, there have been no systematic studies on sweeteners for their effects on the microbiome" seems a little vague and unnecessarily minimizing previous work - is there a better way to specifically highlight the uniqueness of this report?

Response: We have changed the sentence to: "**To date, there has been no study that compared the effect of a comprehensive panel of approved sugar substitute sweeteners on the functionality of *ex-vivo* human gut microbiome**".

13. I'm curious how the authors defined "non-sweetener consumer". The panel included quite a few sweeteners that can be found in a wide range of products. E.g., xylitol is found in many products, like gum, toothpaste, peanut butter / other condiments, at varying levels. Some of these sweeteners also occur naturally in some foods. Was this done in any systematic manner? FFQs? When the volunteers were given a self-assessment on sweetener intake, were they made aware of how prevalent these sweeteners are before determining their own sweetener intake? Were they supplied with a (very extensive) list of items containing these sweeteners?

Response: During volunteer intake and as part of the education for participation in this project and their data collection, the research coordinator counciled the volunteers regarding sweetener intake and the various sources of artificial and naturally occurring sweeteners. All volunteers at intake then completed a nutritional survey to collect patterns of food and nutritional supplement consumption over time. Additionally, at the time of sample collection, a detailed 24-hr diet diary was collected for each volunteer. Based upon these levels of information gathering, those volunteers in this study were defined as "non-sweetener consumers.

Additional comments

Consider using the full names for sweeteners instead of acronyms throughout the text, it would make it much more accessible (as well as make the study identifiable in keyword searches for each sweetener).

Response: We have changed most of the acronyms into full names.

Line 17: Consider changing to "Gut microbiome composition and function are often associated with health and disease status."

Response: We have made the change.

Line 26: Consider rewording this sentence so it is clear exactly what the two clusters are clustering.

Response: We have rewritten the sentence into "**The first "NAS" cluster was composed of non-caloric artificial sweeteners (NAS) and two sugar alcohols with shorter carbon backbones (4-5 carbon atoms), and the second "CHO" (Carbohydrate) cluster was composed of the remaining sugar alcohols**".

Line 30: What do the authors mean by "similar" here? Consider being more specific in the wording so it is clear to the reader exactly what analysis and comparison is being performed.

Response: We have changed the sentence to "**The metaproteomic functional responses of the second cluster were clustered with the prebiotic fructooligosaccharides and kestose**".

Line 37: Consider changing to "The Human gut microbiome"

Response: We have made the change.

Line 68: Missing period after "microbiome".

Response: We have added the period.

Line 109: Briefly define DC assay here in the text

Response: We added the definition.

Line 134: What is "CHO"? Define at first use in the text

Response: The "CHO" refers to "Carbohydrate" and we added the definition.

Line 158 - what are "In addition, proteins with only general function prediction"?

Response: Proteins with general function prediction only is a COG category and we have added the explanation in the revised manuscript.

Line 183 - Use complete names for COG categories

Response: We have changed to use the complete names of COG categories.

Line 251- "clustered sweeteners might promote the cell division of Clostridia." - is the more direct implication of this increased cellular growth?

Response: We have changed the sentence to "**The higher abundance of proteins from these two categories indicated that the "CHO" clustered sweeteners might promote the cellular growth of Clostridia**".

Line 311: "deep-well plates containing pre-reduced, optimized microbiome culture medium" Can the authors describe the type of media used directly here in the text instead of in the reference?

Response: We added the composition of the medium in the revised manuscript.

Line 363-365: Can the authors provide more details on the type of protein search/alignment performed? Were there e-value, percent identity, or mismatch cutoffs required to identify a protein fragment hit? How are protein fragments that map equally well to two different proteins handled? Two different taxa?

Response: We gave more details about peptide/protein identification in the revised manuscript:

"The Metalab software (version 1.2.0) was used for peptide/protein identification and quantification, peptide taxonomy assignment, and protein functional annotation. The searches were performed against a database based on the integrated gene catalog (IGC) which included close-to-complete sets of genes for most gut microbes. **MaxQuant is used as the search engine in MetaLab workflow for peptide/protein identification.** Carbamidomethyl (C) was set as a fixed modification, and protein N-terminal acetylation (protein N-term) and Oxidation (M) were set as variable modifications. **The enzyme was set as trypsin and two missed cleavages were allowed. The false discovery rate (FDR)**

was set as 0.01 in both peptide and protein levels. As one peptide can be matched to multiple proteins. The database search engine will adopt the protein inference method to determine the attribution of peptides."

Line 370- "DESq2" typo.

Response: Fixed.

Line 381 - what database was used for taxonomic assignment, and what taxonomic framework? (NCBI, etc.)

Response: We used a previously constructed pep2tax database to do taxonomy analysis. All the proteins from the non-redundant UniParc database were in silico digested and the corresponding UniProtKB IDs of each protein were used to retrieve the information about taxonomic lineage IDs from UniProtKB database. The constructed pep2tax database was integrated with the MetaLab software (version 1.2.0). We added the reference and more details about the taxonomic assignment in the revised manuscript as below:

"**Taxonomy assignment of each peptide was performed based on the lowest common ancestor (LCA) algorithm. Using a previously constructed pep2tax database integrated with the MetaLab software (version 1.2.0), each identified peptide was assigned to a taxon. The abundance of each taxon was calculated by summarizing the intensities of all distinctive peptides assigned to this taxon**".

Figure 2- "Significantly affected genus" should be "genera" - How is significance determined here? Is it the same as Wilcoxon rank-sum in 2B?

Response: Thank you for pointing out our grammatical errors and it has been fixed. The significance was determined as same as Wilcoxon rank-sum in Fig. 2B and we found that there might be redundancy for the information from previous Fig. 2A and Fig. 2B. So we decided to remove Fig. 2A in the revised version.

Reviewer #2 (Public repository details (Required)):

I confirmed that a dataset has been deposited at ProteomeXchange under the accession number provided. However, access is not public, so I did not confirm the nature of the dataset.

> **Response:** Thank you for providing helpful comments to our manuscript, and we have revised our manuscript accordingly. We hope you will find the revised version satisfactory. In addition, the dataset can now be accessed with a reviewer account (Username: reviewer_pxd030458@ebi.ac.uk, Password: ZZIs0bTv). Once the manuscript is online or published, the dataset will become publicly available.

Reviewer #2 (Comments for the Author):

General comments:

1. It is a little confusing to use two different ways of "counting" the data prior to clustering (genus biomass, and predicted protein function), especially since they gave such different results. I understand why the authors conducted the analyses this way (it gives a microbiota structure and then function flow to the paper), but it does leave the reader wanting to understand how to relate the two analyses. Additionally, the explanation in the Discussion section lines 224 - 228 leaves the reader wondering why the analysis of the microbiota structure using genus biomass was performed at all. At a minimum the genera that are members of class Clostridia could be indicated in lines 122 - 128. Similarly, the genera within class Clostridia that showed differential expression of predicted protein functions could be mentioned in line 181. Maximally, the genus biomass section could be omitted.

> **Response:** Thank you for your suggestions! We did want to have an examination first into the protein biomass of different taxa and then functional profiles as the logic of the paper. Metaproteomics have been used to assess the biomass contributions of different taxa, although it does not directly reflect taxonomic composition as gene sequencing-based techniques, comparison of protein-level biomass can still reflect the overall change of different taxa (Kleiner *et al.*, Nature Communications, 2017). After evaluating the protein biomass, metaproteomics provides a deeper examination into the functionality of these proteins. We first looked at the overall functional category composition at the community level, then examined the taxonomic originations of differential proteins and functions.
>
> We added the indication of the members of class Clostridia in the Figure 2. And in line 181, we also indicated the genera within class Clostridia that showed differential expression of predicted protein functions as below: "**62.6% (134) of the discriminative proteins were from class Clostridia. 46 of the 134 proteins can be assigned to genus level, as they were from 11 genera: *Roseburia* (21), *Clostridium* (6), *Blautia* (4), *Eubacterium* (4), *Lachnoclostridium* (3), *Ruminococcus* (3), *Anaerostipes* (1), *Coprococcus* (1), *Faecalibacterium* (1), *Fusicatenibacter* (1), *Oscillibacter* (1)**".

2. The discussion section is predominantly a restatement of the results. It would be interesting to read more about how these results fit into the existing body of literature on carbohydrate

interactions with the gut microbiota.

Response: We added analysis on CAZyme as described in the next response. And we also added discussion about CAZyme (carbohydrate interactions with the gut microbiota) in the revised manuscript as below:

"**1,075 protein groups were significantly altered by one or more of the sugar substitute sweeteners (Supplementary Table S3). Although it is not possible to assess the possible functional impact of the changes of each protein group, some examples are noteworthy. For example, an average of 54 CAZymes, an important group of enzymes for the microbiome function, were identified per sample, a level consistent with a previous study. Most of the identified CAZymes were glycoside hydrolases and can account for half of the classified CAZmes. A protein group from GH133 (V1.CD38-0_GL0163641) was reduced by 13 of the sugar substitute sweeteners. GH133 CAZymes are glycogen debranching enzymes (alpha-1,6-glucosidase). The decrease in this enzyme class likely indicates that the sugar substitute sweeteners depressed the release of glucose from glycogen storages by some bacteria in the microbiome**".

3. There doesn't seem to be any information on the expression of carbohydrate active enzymes. Is it not possible to get that data from metaproteomes?

Response: It is possible to get information about CAZymes from metaproteomes. We previously did not focus on this aspect, and we added the analysis of CAZyme as described in the method section:

"**The CAZyme (carbohydrate-active enzyme) profile of the microbiome was acquired from the major protein of identified protein groups. The protein sequences of major proteins were extracted from the IGC database and then annotated with dbCAN meta server with two tools, HMMER (E-Value < 1e-15, coverage > 0.35) and DIAMOND (E-Value < 1e-102)**".

The general profile of the CAZymes acquired from metaproteomics was summarized in the result section as below:

"**An average of 54 ± 19 protein groups were identified as CAZyme from each sample. Most of the protein groups were glycoside hydrolases (GH, 40 ± 16 from each sample). Only 25 of 1,075 significantly altered proteins were annotated as CAZyme (Supplementary Table S3). Two protein groups from GH97 (DOM005_GL0010478 and 272559.BF1158), a family comprised of α-glucosidase and α-galactosidase, were significantly increased by hydrogenated starch hydrolysates.**

Specific Comments:

1. Line 134: It seems like CHO is referring to the carbohydrates in that cluster, but I don't see the acronym defined anywhere.

   Response: We added the definition of CHO. It refers to "Carbohydrate".

2. Lines 183 - 184: Please describe the COG categories, rather than stating the letter designation.

   Response: We added the full name of the COG categories.

3. Line 303: "accessed" should be "assessed"

Response: It has been corrected.

4. Line 311: The complete final recipe for the microbiome culture media doesn't seem to be in either of the cited references. It would be very helpful to the reader to add a supplementary table (or some additional text in the methods section) describing the details of the recipe.

Response: Thank you for your suggestion! The medium composition was mentioned in the previous reference 35 (reference 24 in the revised version). And we also added the detail of the final recipe in the revised manuscripts: "**The culture medium was comprised of: 2.0 g L^{-1} peptone water, 2.0 g L^{-1} yeast extract, 0.5 g L^{-1} L-cysteine hydrochloride, 2 mL L^{-1} Tween 80, 5 mg L^{-1} hemin, 10 μL L^{-1} vitamin K1, 1.0 g L^{-1} NaCl, 0.4 g L^{-1} K_2HPO_4, 0.4 g L^{-1} KH_2PO_4, 0.1 g L^{-1} $MgSO_4{\bullet}7H_2O$, 0.1 g L^{-1} $CaCl_2{\bullet}2H_2O$, 4.0 g L^{-1} $NaHCO_3$, 4.0 g L^{-1} porcine gastric mucin, 0.25 g L^{-1} sodium cholate, and 0.25 g L^{-1} sodium chenodeoxycholate**".

April 29, 2022

Dr. Daniel Figeys
University of Ottawa
Ottawa Institute of Systems Biology, Department of Biochemistry, Microbiology and Immunology
Roger Guidon Hall, Room 4207
451 Smyth Rd
Ottawa, ONT K1H 8M5
Canada

Re: Spectrum00412-22R1 (Comprehensive assessment of functional effects of commonly used sugar substitute sweeteners on *ex vivo* human gut microbiome)

Dear Dr. Daniel Figeys:

I apologize for the delay in returning your manuscript - I had some unexpected surgical complications.

Thank you for submitting your manuscript to Microbiology Spectrum. As you will see your paper is very close to acceptance. Please modify the manuscript along the lines I have recommended. As these revisions are quite minor, I expect that you should be able to turn in the revised paper in less than 30 days, if not sooner. If your manuscript was reviewed, you will find the reviewers' comments below.

When submitting the revised version of your paper, please provide (1) point-by-point responses to the issues I raised in your cover letter, and (2) a PDF file that indicates the changes from the original submission (by highlighting or underlining the changes) as file type "Marked Up Manuscript - For Review Only". Please use this link to submit your revised manuscript. Detailed instructions on submitting your revised paper are below.

Link Not Available

Thank you for the privilege of reviewing your work. Below you will find instructions from the Microbiology Spectrum editorial office and comments generated during the review.

The ASM Journals program strives for constant improvement in our submission and publication process. Please tell us how we can improve your experience by taking this quick Author Survey.

Sincerely,

Steven Frese

Editor, Microbiology Spectrum

Comments:

Please carefully review and edit the manuscript for typos, incomplete sentences, and sentence structure.

Additionally, a Reviewer requested additional methods regarding peptide discrimination and mapping (see original query below). The resubmitted text does not full address the Reviewer's query. Please provide additional methodological information.

Original Reviewer Query:
Can the authors provide more details on the type of protein search/alignment performed? Were there e-value, percent identity, or mismatch cutoffs required to identify a protein fragment hit? How are protein fragments that map equally well to two different proteins handled? Two different taxa?

Preparing Revision Guidelines
To submit your modified manuscript, log onto the eJP submission site at https://spectrum.msubmit.net/cgi-bin/main.plex. Go to Author Tasks and click the appropriate manuscript title to begin the revision process. The information that you entered when you first submitted the paper will be displayed. Please update the information as necessary. Here are a few examples of required updates that authors must address:

• point-by-point responses to the issues I raised in your cover letter
• Upload a compare copy of the manuscript (without figures) as a "Marked-Up Manuscript" file.
• Each figure must be uploaded as a separate file, and any multipanel figures must be assembled into one file.
• Manuscript: A .DOC version of the revised manuscript
• Figures: Editable, high-resolution, individual figure files are required at revision, TIFF or EPS files are preferred

For complete guidelines on revision requirements, please see the journal Submission and Review Process requirements at https://journals.asm.org/journal/Spectrum/submission-review-process. **Submissions of a paper that does not conform to Microbiology Spectrum guidelines will delay acceptance of your manuscript.** "

Please return the manuscript within 60 days; if you cannot complete the modification within this time period, please contact me. If you do not wish to modify the manuscript and prefer to submit it to another journal, please notify me of your decision immediately so that the manuscript may be formally withdrawn from consideration by Microbiology Spectrum.

If your manuscript is accepted for publication, you will be contacted separately about payment when the proofs are issued; please follow the instructions in that e-mail. Arrangements for payment must be made before your article is published. For a complete list of **Publication Fees**, including supplemental material costs, please visit ourwebsite.

Corresponding authors may join or renew ASM membership to obtain discounts on publication fees. Need to upgrade your membership level? Please contact Customer Service at Service@asmusa.org.

Thank you for submitting your paper to Microbiology Spectrum.

Comments

Please carefully review and edit the manuscript for typos, incomplete sentences, and sentence structure.

Response: We have carefully revised the manuscript and we hope you find the revised version satisfactory.

Comments

Additionally, a Reviewer requested additional methods regarding peptide discrimination and mapping (see original query below). The resubmitted text does not full address the Reviewer's query. Please provide additional methodological information.

Original query

Can the authors provide more details on the type of protein search/alignment performed? Were there e-value, percent identity, or mismatch cutoffs required to identify a protein fragment hit? How are protein fragments that map equally well to two different proteins handled? Two different taxa?

Response (Black: original manuscript and response; Blue: first revision; Red: second revision, Purple: response):

The MetaLab[1] software (version v1.2.0) which integrated the MaxQuant[2] platform was used for the peptide/protein identification. In proteomics research, the false discovery rate (FDR) is generally used as the filter for peptide and protein identification, and FDR<0.01 was used as the threshold in this study. MaxQuant has given detailed introductions about how the peptide hits are assembled into protein hits[3].

When a peptide is attributed to multiple different species, the last common ancestor (LCA) of the species will be taken as the source of the peptide. And we gave more detailed information in the method section (Microbial taxonomic analysis).

We gave more details about peptide/protein identification in the revised manuscript:

"The Metalab software (version 1.2.0) was used for peptide/protein identification and quantification, peptide taxonomy assignment, and protein functional annotation. The searches were performed against a database based on the integrated gene catalog (IGC) which included close-to-complete sets of genes for most gut microbes. MaxQuant is used as the search engine in MetaLab workflow for peptide/protein identification. Carbamidomethyl (C) was set as a fixed modification, and protein N-terminal acetylation (protein N-term) and Oxidation (M) were set as variable modifications. The enzyme was set as trypsin and two missed cleavages were allowed. The FDR (false discovery rate) was set as 0.01 in both peptide and protein levels. As one peptide can be matched to multiple proteins. The database search engine will adopt the protein inference method to determine the attribution of peptides. Basically, two proteins will join in a protein group when one of the proteins has a set of peptides equal to or completely contained in the other protein. Shared peptides remain in all groups where they occur, but they are most parsimoniously associated with the group that has the

highest number of identified peptides (‘razor’ peptides). Only unique and razor peptides were used for protein quantification. The taxonomy assignment of peptides can be found in the following methods (section Microbial taxonomic analysis). ”

Microbial taxonomic analysis

Taxonomy assignment of each peptide was performed based on the lowest common ancestor (LCA) algorithm. If one peptide is identified in more than one taxon, the peptide will be assigned to the LCA of these taxa. Using a previously constructed pep2tax database integrated with the MetaLab software (version 1.2.0), each identified peptide was assigned to a taxon. The abundance of each taxon was calculated by summarizing the intensities of all distinctive peptides assigned to this taxon. The relative abundance of taxa on a specific taxonomic rank was calculated by normalizing to the summed abundance of all taxa on this rank. For comparison of absolute taxa abundance between samples, relative abundances were multiplied by the total microbial biomass calculated using protein concentration data. Fold changes were calculated between sweetener-treated samples and PBS control from the same microbiome.

References

(1) Cheng, K.; Ning, Z.; Zhang, X.; Li, L.; Liao, B.; Mayne, J.; Stintzi, A.; Figeys, D. MetaLab: An Automated Pipeline for Metaproteomic Data Analysis. *Microbiome* **2017**, *5* (1), 157. https://doi.org/10.1186/s40168-017-0375-2.

(2) Tyanova, S.; Temu, T.; Cox, J. The MaxQuant Computational Platform for Mass Spectrometry-Based Shotgun Proteomics. *Nat Protoc* **2016**, *11* (12), 2301–2319. https://doi.org/10.1038/nprot.2016.136.

(3) Cox, J.; Mann, M. MaxQuant Enables High Peptide Identification Rates, Individualized p.p.b.-Range Mass Accuracies and Proteome-Wide Protein Quantification. *Nat Biotechnol* **2008**, *26* (12), 1367–1372. https://doi.org/10.1038/nbt.1511.

May 21, 2022

Dr. Daniel Figeys
University of Ottawa
Ottawa Institute of Systems Biology, Department of Biochemistry, Microbiology and Immunology
Roger Guidon Hall, Room 4207
451 Smyth Rd
Ottawa, ONT K1H 8M5
Canada

Re: Spectrum00412-22R2 (Comprehensive assessment of functional effects of commonly used sugar substitute sweeteners on *ex vivo* human gut microbiome)

Dear Dr. Daniel Figeys:

Your manuscript has been accepted, and I am forwarding it to the ASM Journals Department for publication. You will be notified when your proofs are ready to be viewed.

The ASM Journals program strives for constant improvement in our submission and publication process. Please tell us how we can improve your experience by taking this quick Author Survey.

As an open-access publication, Spectrum receives no financial support from paid subscriptions and depends on authors' prompt payment of publication fees as soon as their articles are accepted. You will be contacted separately about payment when the proofs are issued; please follow the instructions in that e-mail. Arrangements for payment must be made before your article is published. For a complete list of **Publication Fees**, including supplemental material costs, please visit ourwebsite.

ASM policy requires that data be available to the public upon online posting of the article, so please verify all links to sequence records, if present, and make sure that each number retrieves the full record of the data. If a new accession number is not linked or a link is broken, provide production staff with the correct URL for the record. If the accession numbers for new data are not publicly accessible before the expected online posting of the article, publication of your article may be delayed; please contact the ASM production staff immediately with the expected release date.

Corresponding authors may join or renew ASM membership to obtain discounts on publication fees. Need to upgrade your membership level? Please contact Customer Service at Service@asmusa.org.

Thank you for submitting your paper to Spectrum.

Sincerely,

Steven Frese
Editor, Microbiology Spectrum

Journals Department
American Society for Microbiology
1752 N St., NW
Washington, DC 20036
E-mail: spectrum@asmusa.org

Supplemental file2: Accept
Supplemental file1: Accept
